# Disease control and its correlates among hypertensive, diabetic, and asthmatic patients enrolled in secondary-level Integrated Chronic Care Clinics in Neno District, rural Malawi

**DOI:** 10.1371/journal.pgph.0006629

**Published:** 2026-07-30

**Authors:** George Dalitso Limwado, Beatrice Lydia Matanje, Victor Chigonjetso Mithi, Francis Valeta, Matamando Mwendera, Bright Ngongonda, Kondwani Matiya, Owen Mwale, Henry Chimuso Phiri, Enoch Ndarama, Limbani Thengo, Moses Banda Aron, Kondwani Mpinga, Luckson Dullie

**Affiliations:** 1 Partners in Health/ Abwenzi Pa Za Umoyo, Neno, Malawi; 2 National AIDS Commission, Lilongwe, Malawi; 3 The University of Global Health Equity, Kigali, Rwanda; 4 Ministry of Health, Neno District Health Office, Neno District Hospital, Neno, Malawi; Faculty of Medicine, Universitas Sebelas Maret, INDONESIA

## Abstract

Non-communicable diseases (NCDs) account for nearly 74% of global deaths, with 86% of premature NCD mortality occurring in low- and middle-income countries. In Malawi, hypertension, diabetes mellitus, and asthma contribute substantially to morbidity and mortality. Integrated service delivery models such as the Integrated Chronic Care Clinics (IC3) in Neno District aim to improve chronic disease management; however, evidence on disease control within these models remains limited. This study assessed disease control and associated correlates among patients receiving care in IC3 clinics in rural Malawi. We conducted a mixed-methods retrospective cross-sectional study at Neno District Hospital and Lisungwi Community Hospital. Quantitative data were extracted from OpenMRS electronic medical records for 1,790 patients diagnosed with hypertension, diabetes, or asthma. Disease control was defined as blood pressure <140/90 mmHg (<130/80 mmHg for hypertensive diabetics), HbA1c < 7% for diabetes, and symptom-based criteria for asthma. Associations between disease control and demographic or clinical variables were assessed using chi-square tests and logistic regression. Qualitative data were collected through 17 focus group discussions and 56 in-depth interviews with patients and healthcare providers. Hypertension control was achieved in 55% of patients, asthma control in 87%, and diabetes control in 32%. Males had lower odds of hypertension control than females (aOR 0.70, 95% CI 0.55–0.90). Overweight patients and those receiving more than two antihypertensive medications also had lower odds of hypertension control. Asthma control was associated with inhaled corticosteroid use, while glycaemic control was associated with the number of prescribed medications. Qualitative findings identified health literacy, medication adherence, patient–provider communication, and access to care as key influences on disease management. Disease control varied across conditions within integrated chronic care clinics in rural Malawi and was influenced by demographic, treatment-related, and contextual factors.

## Introduction

Non-communicable diseases (NCDs) remain the leading cause of death worldwide, accounting for approximately 60% of global deaths, equivalent to about 35 million people annually [1,2]. NCDs also contribute substantially to premature mortality, with more than 15 million deaths occurring among individuals aged 30–69 years [1]. The growing burden of NCDs poses significant challenges for health systems in low- and middle-income countries (LMICs), which account for approximately 86% of premature NCD-related deaths and 77% of the global NCD mortality burden [3,4]. In addition to their health impact, NCDs have major economic consequences [5]. Globally, they are projected to result in a cumulative economic loss of approximately US$47 trillion between 2011 and 2030, potentially deepening poverty and slowing development in LMICs [4,5]. Addressing this burden requires effective prevention and long-term management strategies, particularly in settings where health systems face competing priorities and limited resources.

Although communicable diseases currently contribute a larger share of mortality in sub-Saharan Africa, projections suggest that by 2030, NCDs will surpass communicable diseases as the leading cause of death in the region [3,5]. Malawi is already experiencing this epidemiological transition [6]. NCDs account for approximately 28% of all deaths nationally, exceeding the annual mortality attributed to Human Immunodeficiency Virus (HIV) [6]. This increasing burden is driven by several factors, including urbanisation and associated lifestyle changes, increasing life expectancy, and the complex interactions between communicable diseases and chronic conditions [6]. In addition to mortality, NCDs contribute significantly to disability and long-term morbidity. A regional analysis reported a 67% increase in disability-adjusted life years (DALYs) attributable to NCDs in sub-Saharan Africa between 1990 and 2017 [3].

Globally, the most common NCDs include cardiovascular diseases (CVDs), chronic respiratory diseases, cancers, mental health disorders, and diabetes mellitus [1,2]. Similar patterns are observed across much of sub-Saharan Africa, including Malawi, where cancer is the leading NCD, followed by cardiovascular diseases [7–12]. Hypertension is the most prevalent cardiovascular condition in the country, with estimates ranging from 15.8% to 32.9%, and is a major contributor to cardiovascular morbidity and mortality [6,9–11]. Diabetes mellitus prevalence in Malawi ranges from approximately 1.7% to 5.6%, while asthma prevalence has been estimated between 4.5% and 5.1% [8,9,11,13,14]. Effective management of these chronic conditions requires sustained clinical monitoring, access to medications, and strong patient engagement with care services.

In response to the growing NCD burden, several global initiatives have been developed to support prevention and management in resource-limited settings. In 2010, the World Health Organization (WHO) introduced the Package of Essential Non-communicable Disease Interventions (PEN) for primary care in low-resource settings [14,15]. This set of cost-effective interventions aims to strengthen the management of priority NCDs through simplified clinical protocols and decentralised service delivery. Building on this framework, WHO later launched PEN Plus, which focuses on decentralising specialised NCD care, traditionally delivered at tertiary facilities, to secondary-level hospitals in order to improve access to diagnosis and treatment and reduce complications [15,16].

An additional strategy for strengthening NCD care in resource-limited settings involves integrating NCD services with existing chronic disease platforms. In Malawi, Partners In Health/Abwenzi Pa Za Umoyo (PIH/APZU) implemented such an approach in Neno District through the Integrated Chronic Care Clinics (IC^3^), where NCD services are delivered alongside HIV care [17]. This model leverages clinical infrastructure, human resources, and monitoring systems originally developed for HIV programmes. Evidence from Neno District suggests that integrating NCD and HIV services can improve access to care, reduce stigma associated with HIV services, and support retention in long-term treatment programmes [17–19]. The IC^3^ model also creates a “one-stop” service platform for individuals living with multiple chronic conditions, potentially improving continuity of care and overall treatment coordination [18–20].

Integrated chronic care models are hypothesised to influence disease outcomes through several pathways. By consolidating services within a single clinic platform, integrated care may improve continuity of care, strengthen patient–provider relationships, and enhance clinical monitoring. These mechanisms may support improved disease control through increased medication adherence, better patient knowledge and self-management, and more consistent access to essential medicines [21–24]. Within such models, disease outcomes are shaped by a combination of patient-level factors (such as demographic characteristics and health literacy), treatment-related factors (including medication type and regimen complexity), and health system factors (such as accessibility of services and quality of patient–provider communication) [21,23,24].

Although integrated chronic care models are increasingly implemented across sub-Saharan Africa, empirical evidence describing disease control outcomes and associated factors within these models remains limited, particularly in rural district health systems. Most studies in the region focus on individual diseases rather than examining multiple chronic conditions within integrated care platforms. Furthermore, there is limited evidence from Malawi describing factors associated with disease control among patients enrolled in integrated NCD care programmes.

Therefore, this study aimed to assess the extent of disease control among patients with hypertension, diabetes, and asthma receiving care in IC^3^ in Neno District, Malawi, and to examine demographic, clinical, and treatment-related factors associated with disease control. Given the limited prior evidence from integrated care settings in rural Malawi, the quantitative analyses were primarily exploratory and intended to identify potential correlates of disease control, while qualitative data were used to contextualise patient and provider perspectives on chronic disease management within the IC^3^ model.

## Methodology

### Ethical considerations

This study was conducted with the approval of the Neno District Health Research Committee (NDHRC) and the National Health Science Research Committee (NHSRC) in Malawi under protocol number 23/04/4063, titled “Evaluation of Clinical Care in PIH/APZU-supported Sites in Malawi.” All retrospective data used in this study were fully anonymised before access and analysis by the research team. As such, the ethics committees waived the requirement for informed consent for the review of medical records. However, for any components of the study that involved direct participant interaction, such as interviews or focus group discussions (FGDs), written informed consent was obtained from participants, with a witness present, in accordance with ethical guidelines.

### Study design

We conducted a mixed-method, retrospective cross-sectional study from 3^rd^ July to 22^nd^ December 2023, to evaluate the care outcomes of individuals receiving treatment for hypertension, diabetes mellitus, and asthma at the IC^3^. During this period, patient records were reviewed to collect data on disease management, treatment adherence, and clinical outcomes related to these prevalent NCDs. Additionally, recipients of care (ROCs) participated in individual interviews and focus group discussions to provide qualitative insights into their experiences and perspectives on disease management and healthcare services.

### Study setting

The study was conducted in Neno District, located in the southwestern part of Malawi. Covering a total land area of 1,561 square kilometres, Neno District accounts for approximately 1.6% of the total land area of Malawi [25,26]. According to the 2018 Malawi Population and Housing Census, the district has a population of 153,132 [27,28]. The district is served by fifteen health facilities, with Lisungwi Community Hospital and Neno District Hospital functioning as the referral secondary care hospitals for the district [29]. This study specifically took place at Neno District Hospital (NDH) and Lisungwi Community Hospital (LCH) IC^3^.

### Sampling method

#### Sample size.

A consecutive sampling approach was used, including all eligible recipients of care (ROCs) meeting the inclusion criteria during the study period. The sample size for the qualitative component was determined iteratively based on thematic saturation. For the quantitative component, the Cochrane formula was used based on the population of ROCs with hypertension, diabetes mellitus, and asthma, to guide the number of participants required for interviews [30].

### Participant recruitment

Participants for the qualitative component were recruited during routine clinic visits using a systematic sampling approach. On each clinic day, eligible recipients of care (ROCs) were identified from the clinic register, and every nth ROC (based on clinic flow) was approached for participation to ensure variation across patient characteristics and reduce selection bias. Where selected individuals declined participation, the next eligible patient was approached.

The aims and objectives of the study were explained verbally, and all participants were informed that participation was voluntary and that their responses would remain anonymous and confidential. Written informed consent was obtained from those willing to participate. Recruitment continued on each clinic day until the required sample size was reached. Focus group discussions (FGDs) and in-depth interviews (IDIs) were conducted iteratively, and data collection continued until thematic saturation was achieved, defined as the point at which no new themes emerged from successive interviews.

### Inclusion criteria

#### Qualitative component.

Participants were required to be 18 years or older, diagnosed with diabetes, hypertension, or asthma, and receiving care through IC^3^ between January 2017 and December 2021. Only those who provided informed consent and had been on treatment for at least six months prior to data collection were included.

#### Quantitative component.

Inclusion criteria. All adult ROCs diagnosed with the same conditions and actively enrolled in care at NDH and LCH between January 2017 and December 2021 were included, regardless of the duration of their treatment. Electronic medical records were accessible from the 3^rd^ of July to the 31^st^ of July 2023, and we recruited ROCs from the 7^th^ to the 31^st^ of August 2023, for interviews and FGDs.

Exclusion criteria. ROCs who did not consent to participate in the study, as well as those who had been in care before January 2017 or after December 2021, were excluded from the study. Individuals with type 1 diabetes mellitus or those requiring insulin therapy were excluded from the analysis because, within the IC^3^ model in Neno District, such patients are followed under the Advanced NCD Clinic rather than the standard IC^3^ program assessed in this study.

### Data collection

#### Quantitative component.

Data was extracted from the OpenMRS electronic medical records systems (EMR). The system captures and stores data for patients attending routine clinical care for NCDs at all health facilities in Neno District. Data extraction had filters for health facilities of the NCD program enrollment at NDH and LCH in rural Malawi. We developed a questionnaire by reviewing the available literature [7–11,13–16,31–35]. A comprehensive literature review was conducted using databases such as PubMed and Google Scholar, focusing on terms such as “Hypertension,” “Diabetes Mellitus,” “Asthma,” “Sub-Saharan Africa”, and “Malawi”. These tools were in English, translated into Chichewa (Malawi’s official local language), and then back-translated into English to ensure accuracy in the translation process.

#### Measurements.

Clinical measurements were also taken on the day of the interview, including:

- Blood Pressure: Measured using an electronic sphygmomanometer, recorded in millimetres of mercury (mmHg).
- Body Mass Index (BMI): Assessed using an adult weighing scale, recorded in kilograms (kg) and height in meters, then BMI was calculated using the formula: Mass/height^2^.
- HbA1c Levels: Obtained through point-of-care testing to evaluate diabetes control.
- Asthma Attacks: The number of asthma attacks experienced by participants in the previous week and month was documented.

We defined disease control as follows:

- According to the American Diabetes Association, patients with Diabetes Mellitus are classified into two groups: those whose HbA1c levels are maintained above 7% are considered uncontrolled, while those with HbA1c levels below 7% are classified as controlled [8,36].
- For hypertension, control is defined as a blood pressure reading below 140/90 mm Hg; for hypertensive patients with diabetes comorbidity, the threshold is set at below 130/80 mm Hg [9,31].
- Regarding asthma, a patient is considered to have controlled asthma if they report symptoms and use reliever medication less than twice a week, experience no nocturnal symptoms, have no activity limitations, and lack significant risk factors, such as a history of intubation [34,35].

#### Qualitative component.

ROCs were approached during routine clinic visits and invited to participate in in-depth interviews (IDIs) or focus group discussions (FGDs). After providing written informed consent, interviews were scheduled at a convenient time and conducted in a private location within the health facility to ensure confidentiality and allow participants to speak freely. Interviews were conducted in Chichewa (the local language) by trained members of the research team who were experienced in qualitative data collection within the Neno IC^3^ programme. All interviews were audio-recorded with participants’ permission and typically lasted between 30 and 60 minutes.

Semi-structured interview and FGD guides were initially developed in English and subsequently translated into Chichewa. The guides were pretested at Zalewa and Ligowe IC^3^ clinics to assess clarity, cultural appropriateness, and feasibility. Based on feedback from pilot testing and consultation with members of the research team, minor revisions were made to improve the structure and wording of the questions.

Following pilot testing, FGDs were conducted with groups of patients stratified by type of NCD and disease control status (controlled versus uncontrolled) to facilitate discussion of shared experiences. The qualitative component explored several domains related to chronic disease management within the IC^3^ model, including demographic and socio-economic context, patient knowledge of disease and treatment, duration of care, and experiences with treatment and follow-up services.

Discussions also explored perceived factors influencing disease control across four broad domains: (1) patient-related factors, including beliefs, attitudes, and health behaviours; (2) treatment-related factors, such as medication regimens, adherence, and side effects; (3) illness-related factors, including perceived severity and disease progression; and (4) health system factors, including accessibility of services, availability of medications, and quality of patient–provider communication.

### Statistical analysis

Quantitative data were extracted from the PIH/APZU electronic medical records (EMR) system into Microsoft Excel for cleaning and subsequently analysed using STATA version 18.0. Categorical variables, including sex, marital status, educational level, age group, disease duration, income, follow-up adherence, and presence of complications, were summarised using frequencies and proportions.

Bivariate analyses were conducted to assess associations between disease control status and selected independent variables using the Chi-square test, we used Cramer’s *V* to measure the strength of association [37].

Multivariable logistic regression was performed for hypertension outcomes to estimate adjusted odds ratios (aORs) and 95% confidence intervals for factors associated with blood pressure control [38]. The multivariable model included demographic and treatment-related variables identified from prior literature and bivariate analysis [9,10]. Due to smaller subgroup sizes for diabetes and asthma, multivariable modelling for these conditions was not performed to avoid unstable estimates, and results for these conditions are presented descriptively.

For the qualitative component, audio-recorded interviews and focus group discussions were transcribed verbatim by members of the research team. When interviews were conducted in Chichewa, transcripts were translated into English using a forward- and back-translation process to ensure accuracy. Transcripts were then uploaded into Dedoose qualitative analysis software.

Qualitative data were analysed using thematic analysis. Audio recordings were transcribed verbatim in Chichewa and translated into English. To ensure accuracy, selected transcripts were cross-checked against the original recordings by members of the research team.

The analytic process began with open coding, during which transcripts were reviewed line-by-line to identify initial concepts and categories emerging from the data. A preliminary coding framework was developed inductively from the data and applied to subsequent transcripts. Through axial coding, relationships between categories were examined, and broader themes were developed.

Coding and theme development were conducted collaboratively by members of the research team to enhance analytic rigour. Differences in interpretation were discussed and resolved through consensus, and the coding framework was iteratively refined throughout the analysis process. Data collection and analysis occurred concurrently, allowing emerging themes to inform subsequent interviews. Recruitment continued until thematic saturation was reached, defined as the point at which additional interviews did not yield new themes or substantive insights.

Given that several members of the research team were clinicians or programme staff involved in NCD care delivery, reflexive discussions were held during analysis to consider how prior experiences and perspectives might influence interpretation of the data. These discussions helped ensure that the analysis remained grounded in participants’ narratives rather than researchers’ assumptions

## Results

The study included 1,790 unique recipients of care. Because some participants had more than one diagnosis, the total number of conditions exceeded the number of individuals. Hypertension was the most common condition (n = 1,301), followed by asthma (n = 451) and diabetes (n = 87). A proportion of participants had multimorbidity, including coexisting hypertension with diabetes or asthma.

### Participant characteristics

Among individuals with asthma (n = 451), the majority were female (n = 276, 61.2%), and most were younger than 50 years of age (n = 359, 79.6%). Regarding nutritional status, 41.2% were overweight, and 26.2% were underweight. Over half of the participants reported a family history of asthma (56.5%), and most were HIV-negative (96.0%). The majority had no additional comorbid conditions (86.7%). Slightly more than half of asthma patients (54.8%) were receiving a single medication. Overall, 87.4% of individuals with asthma had controlled disease.

Among participants with diabetes (n = 87), slightly more than half were male (56.3%), and 60.9% were aged 50 years or older. Most participants were overweight (72.4%), while only a small proportion were underweight (3.4%). Approximately one-third had coexisting hypertension (34.5%), and 6.9% were living with HIV. Over half of the participants (58.6%) were receiving two types of medications. Glycaemic control was suboptimal, with 67.8% of participants classified as having uncontrolled diabetes.

Among hypertensive participants (n = 1,301), the majority were female (69.0%), and most were aged 50 years or older (77.4%). Nearly half were overweight (47.0%), while 12.7% were underweight. Approximately one-quarter reported a family history of hypertension (26.5%), and 9.7% were living with HIV. Most participants had no additional comorbid conditions (81.9%). About treatment, 46.3% were taking two antihypertensive medications, and 36.4% were receiving more than two types of drugs. Overall, 54.7% of hypertensive participants had controlled blood pressure (Table 1)

**Table 1:** Demographic characteristics of ROC examined in the study.

Demographics			
	**Diabetes**	**Asthma**	**Hypertension**
	n = 87	n = 451	n = 1,301
Sex			
Female	38 (43.7%)	276 (61.2%)	898 (69.0%)
Male	49 (56.3%)	175 (38.8%)	403 (31.0%)
Age Category			
< 50	34 (39.1%)	359 (79.6%)	294 (22.6%)
>=50	53 (60.9%)	92 (20.4%)	1,007 (77.4%)
BMI			
Underweight	3 (3.4%)	118 (26.2%)	165 (12.7%)
Normal	21 (24.1%)	147 (32.6%)	524 (40.3%)
Overweight	63 (72.4%)	186 (41.2%)	612 (47.0%)
Family History			
Yes	22 (25.3%)	255 (56.5%)	345 (26.5%)
No	50 (57.5%)	59 (13.1%)	593 (45.6%)
Unknown	15 (17.2%)	137 (30.4%)	363 (27.9%)
HIV			
Yes	6 (6.9%)	18 (4.0%)	126 (9.7%)
No	81 (93.1%)	433 (96.0%)	1,175 (90.3%)
Comorbidity			
None	6 (6.9%)	391 (86.7%)	1,066 (81.9%)
One	51 (58.6%)	57 (12.6%)	225 (17.3%)
Two	30 (34.5%)	3 (0.7%)	10 (0.8%)
Drugs			
One	36 (41.4%)	247 (54.8%)	225 (17.3%)
Two	51 (58.6%)	204 (45.2%)	603 (46.3%)
More than two types	N/A	N/A	473 (36.4%)
Hypertension Control			
Controlled	N/A	N/A	712 (54.7%)
Uncontrolled	N/A	N/A	589 (45.3%)
Asthma Control			
Controlled	N/A	394 (87.4%)	N/A
Uncontrolled	N/A	57 (12.6%)	N/A
Diabetes Control			
Controlled	28 (32.2%)	N/A	N/A
Uncontrolled	59 (67.8%)	N/A	N/A
Asthma Diagnosis			
Yes	1 (1.1%)	N/A	57 (4.4%)
No	86 (98.9%)	N/A	1,244 (95.6%)
Diabetes Diagnosis			
Yes		2 (0.4%)	62 (4.8%)
No		449 (99.6%)	1,239 (95.2%)
Hypertension Diagnosis			
Yes	30 (34.5%)	43 (9.5%)	N/A
No	57 (65.5%)	408 (90.5%)	N/A

### Factors associated with disease control (bivariate analysis)

Bivariate analysis showed no statistically significant associations between asthma control and sex, age category, BMI, family history, HIV status, or comorbidity status (p > 0.05). However, the number of medications used was significantly associated with asthma control (p < 0.001), with higher control observed among individuals receiving a single medication.

Similarly, for diabetes, no significant associations were observed between glycaemic control and demographic or clinical factors such as sex, age category, BMI, family history, HIV status, or comorbidity status (p > 0.05). However, the number of medications prescribed showed a statistically significant association with diabetes control (p = 0.040).

Among individuals with hypertension, several factors were significantly associated with blood pressure control in bivariate analysis. Sex was associated with control status, with females more likely to have controlled hypertension than males (p = 0.005). Body mass index was also associated with control (p = 0.005), as was the number of medications taken (p < 0.001). Family history of hypertension showed a modest association with control status (p = 0.017). No significant associations were observed between hypertension control and age category, HIV status, or comorbidity status (Table 2).

**Table 2:** Factors associated with disease control.

Bivariate Analysis									
	**Diabetes Control**			**Asthma Control**			**Hypertension Control**		
	**Uncontrolled**	**Controlled**	**p-value (Crude OR; CI)**	**Uncontrolled**	**Controlled**	**p-value (Crude OR; CI)**	**Uncontrolled**	**Controlled**	**p-value (Crude OR; CI)**
Sex									
Female	26 (44.1%)	12 (42.9%)	0.915 (1.051; 0.387, 2.898)	29 (50.9%)	247 (62.7%)	0.087 (0.616; 0.340)	383 (65.0%)	515 (72.3%)	0.005 (0.711; 0.558, 0.907)
Male	33 (55.9%)	16 (57.1%)		28 (49.1%)	147 (37.3%)		206 (35.0%)	197 (27.7%)	
Age Category									
< 50	24 (40.7%)	10 (35.7%)	0.658 (1.234; 0.445, 3.539)	42 (73.7%)	317 (80.5%)	0.236 (0.680; 0.348, 1.393)	131 (22.2%)	163 (22.9%)	0.780 (0.963; 0.735, 1.261)
>=50	35 (59.3%)	18 (64.3%)		15 (26.3%)	77 (19.5%)		458 (77.8%)	549 (77.1%)	
BMI									
Underweight	2 (3.4%)	1 (3.6%)	0.129 (0.947; 0.047, 57.931)	15 (26.3%)	103 (26.1%)	0.321 (1.001; 0.498, 1.952)	61 (10.4%)	104 (14.6%)	0.005 (0.675; 0.474, 0.957)
Normal	18 (30.5%)	3 (10.7%)		14 (24.6%)	133 (33.8%)		224 (38.0%)	300 (42.1%)	
Overweight	39 (66.1%)	24 (85.7%)		28 (49.1%)	158 (40.1%)		304 (51.6%)	308 (43.3%)	
Family History									
Yes	15 (25.4%)	7 (25.0%)	0.994 (1.020; 0.369, 2.777)	32 (56.1%)	223 (56.6%)	0.111 (1.969; 0.882, 4.121)	167 (28.4%)	178 (25.0%)	0.017 (0.726; 0.579, 0.911)
No	34 (57.6%)	16 (57.1%)		12 (21.1%)	47 (11.9%)		243 (41.3%)	350 (49.2%)	
Unknown	10 (16.9%)	5 (17.9%)		13 (22.8%)	124 (31.5%)		179 (30.4%)	184 (25.8%)	
HIV									
Yes	3 (5.1%)	3 (10.7%)		1 (1.8%)	17 (4.3%)		63 (10.7%)	63 (8.8%)	0.262 (0.810; 0.552, 1.191)
No	56 (94.9%)	25 (89.3%)	0.333 (2.240; 0.277, 17.739)	56 (98.2%)	377 (95.7%)	0.356 (2.525; 0.380, 107.373)	526 (89.3%)	649 (91.2%)	
Comorbidity									
None	3 (5.1%)	3 (10.7%)	0.433 (0.446; 0.056, 3.604)	53 (93.0%)	338 (85.8%)	0.118 (2.195; 0.762, 8.666)	468 (79.5%)	598 (84.0%)	0.052 (0.737; 0.550, 0.989)
One	37 (62.7%)	14 (50.0%)		3 (5.3%)	54 (13.7%)		114 (19.4%)	111 (15.6%)	
Two	19 (32.2%)	11 (39.3%)		1 (1.8%)	2 (0.5%)		7 (1.2%)	3 (0.4%)	
Drugs									
One	20 (33.9%)	16 (57.1%)	0.040 (0.384; 0.138, 1.066)	9 (15.8%)	238 (60.4%)	<0.001 (0.122; 0.517, 0.264)	82 (13.9%)	143 (20.1%)	<0.001 (0.644; 0.472, 0.874)
Two	39 (66.1%)	12 (42.9%)		48 (84.2%)	156 (39.6%)		228 (38.7%)	375 (52.7%)	
More than two types	N/A	N/A		N/A	N/A		279 (47.4%)	194 (27.2%)	

### Multivariate analysis

Multivariable logistic regression analysis was conducted for hypertension to identify independent predictors of blood pressure control.

Male participants had significantly lower odds of achieving blood pressure control compared with females (adjusted odds ratio [aOR] = 0.71; 95% CI: 0.55–0.91; p = 0.006). Being overweight was also independently associated with lower odds of hypertension control (aOR = 0.56; 95% CI: 0.39–0.81; p = 0.002) compared with being underweight Table 3.

**Table 3:** Multivariate analysis of factors associated with disease control among hypertensive ROC.

Multivariate Analysis			
	**Hypertension Control**		
Variable	aOR	p-value	95% CI
Sex			
Female	*ref*	*ref*	*ref*
Male	0.708	0.006	[0.553, 0.907]
Age Category			
< 50	*ref*	*ref*	*ref*
>=50	0.967	0.811	[0.738, 1.269]
BMI			
Underweight	*ref*	*ref*	*ref*
Normal	0.785	0.198	[0.542, 1.135]
Overweight	0.560	0.002	[0.389, 0.805]
HIV			
No	*ref*	*ref*	*ref*
Yes	1.103	0.727	[0.635, 1.915]
Comorbidity			
None	*ref*	*ref*	*ref*
One	0.771	0.228	[0.505, 1.177]
Two	0.509	0.316	[0.136, 1.906]
Drugs			
One	*ref*	*ref*	*ref*
Two	0.978	0.892	[0.710, 1.347]
More than two types	0.410	0.000	[0.294, 0.571]

Participants receiving more than two antihypertensive medications had significantly lower odds of achieving blood pressure control compared with those receiving a single medication (aOR = 0.41; 95% CI: 0.29–0.57; p < 0.001). No statistically significant associations were observed between hypertension control and age category, HIV status, or comorbidity status.

A total of 179 participants (143 females and 36 males) contributed to the qualitative component through 17 focus group discussions (FGDs) and 56 in-depth interviews (IDIs). Among IDI participants, 14 had hypertension, 13 had asthma, and 13 had diabetes, while one participant reported both hypertension and asthma.

Thematic analysis using Dedoose identified four principal themes: (1) health literacy and disease knowledge, (2) access to healthcare, (3) patient–provider communication and education, and (4) self-care and health-seeking behaviour. For diabetes, IDI data generated 20 codes aligned with these themes, indicating consistency in thematic structure across conditions.

### Health literacy and disease knowledge

Participants demonstrated varying levels of understanding regarding the causes, triggers, and management of their conditions. Several individuals with asthma identified hereditary predisposition as a risk factor:

“Asthma is indeed genetic and not contagious.” (FGD, asthma uncontrolled)

Some participants with hypertension and diabetes described lifestyle-related contributors, including diet:

“Sometimes it is also caused by eating more fatty foods, which makes the heart not function properly.” (FGD, hypertension controlled)

Participants with diabetes also reported perceived improvements following treatment initiation:

“I saw it because that time I didn’t see properly, but when I started receiving the medication, I saw changes in my sight.” (IDI, diabetes controlled)

Environmental triggers such as cold weather and smoke were commonly reported among asthma participants:

“When it’s cold, asthma is triggered, and it makes you have difficulties in breathing.” (FGD, asthma uncontrolled)

Misconceptions were also identified, including beliefs that asthma is contagious:

“Asthma is contagious; you can transmit it to someone else… if you are eating from the same plate.” (FGD, asthma uncontrolled)

### Access to healthcare

Participants frequently described logistical challenges in accessing care, including long travel distances and transport costs:

“I walk on foot, but when I have money, I use a bike; to and from costs MK 700.” (IDI, asthma controlled)

“I walk a distance of about 9 km.” (FGD, diabetes controlled)

Patient–provider communication and education

Most participants reported positive interactions with healthcare providers and expressed trust in the care received:

“I don’t know why some people hate hospital treatment; they are ruining their future because they do not want to go to the hospital.” (FGD, asthma uncontrolled)

“I can’t lie, when I arrive here, everything goes smoothly. They welcome us and attend to us in the right way.” (IDI, diabetes controlled)

However, some participants described challenges in adhering to lifestyle recommendations:

“Some patients don’t like to be given medical advice… especially being told to stop drinking alcohol.” (FGD, hypertension uncontrolled)

### Self-care and health-seeking behaviour

Participants described a range of self-management practices, including medication adherence, dietary modification, and avoidance of triggers:

“I take my medication in the morning… and avoid food with too much salt, sugar, or oil.” (IDI, hypertension controlled)

“I follow the advice from the hospital that I should take my medication morning and evening.” (IDI, diabetes controlled

## Discussion

In this study, we assessed correlates of disease control among ROCs with asthma, diabetes mellitus, and hypertension within the IC^3^ in Neno District, rural Malawi. To our knowledge, this is the first comprehensive evaluation since IC^3^’s inception in 2015 to examine both clinical outcomes and patient perspectives systematically. This work is particularly important given that, while IC^3^ has been recognised for its innovation in integrating NCD and HIV care within PIH/APZU–supported health systems, specific correlates within the model associated with improved disease outcomes have not been fully characterized [17,18]. Our findings provide important insights that can help strengthen clinical practice, inform targeted programmatic improvements, and guide the adaptation of integrated chronic care models in similar resource-limited settings

Overall, the findings suggest that disease control was variably associated with medication regimens and certain demographic characteristics, while many demographic and clinical variables were not significantly associated with control status. Importantly, the analysis was not designed to evaluate the effectiveness of the IC^3^ model but rather to identify correlates of disease control among patients receiving care within this system.

Sex differences were observed for hypertension control, where male participants had significantly lower odds of achieving blood pressure control compared with female participants. This finding is consistent with evidence from other low- and middle-income countries suggesting that men may be less likely to engage consistently with chronic disease care or adhere to long-term treatment regimens [10,39–43]. Qualitative findings from this study support this interpretation, as some participants described reluctance among certain patients to follow lifestyle recommendations such as reducing alcohol consumption or modifying dietary habits. However, alternative explanations should also be considered. For example, differential care-seeking behaviour may influence which patients remain engaged in clinic-based care. Women in many rural settings have more frequent interactions with health services through reproductive and child health programmes, potentially increasing opportunities for screening and follow-up [39,44,45]. Conversely, men who remain engaged in care may represent a subgroup with more advanced disease or more complex clinical needs, which could contribute to lower observed control rates [39]. These considerations highlight the importance of gender-sensitive approaches to chronic disease management, particularly strategies aimed at improving male engagement in long-term care.

BMI was also associated with hypertension control, with overweight individuals demonstrating lower odds of achieving blood pressure control compared with underweight participants. This finding is consistent with established evidence linking excess body weight with poorer cardiovascular outcomes and greater difficulty achieving blood pressure targets [10,40]. However, the relationship between BMI and disease control in this study may also reflect unmeasured behavioural and metabolic factors, including dietary patterns, physical activity levels, and comorbid metabolic conditions [42,45]. Qualitative data highlighted patient awareness of dietary risk factors such as high salt and fat consumption, suggesting that lifestyle behaviours may play a role in disease management. At the same time, BMI categories were derived from routine clinical measurements and may not fully capture body composition or metabolic risk. Therefore, while the observed association aligns with existing literature, it should be interpreted cautiously and considered within the broader behavioural and clinical context influencing hypertension management [42,44,45].

For asthma, demographic and clinical characteristics such as sex, age, BMI, and family history were not significantly associated with disease control in quantitative analyses. Instead, medication use appeared to be the strongest correlate of asthma control, with significant differences observed across treatment regimens. These findings are consistent with clinical evidence demonstrating the effectiveness of inhaled corticosteroids and bronchodilators in improving asthma control [35,46–48]. Qualitative findings provide additional context, as participants frequently described environmental triggers such as cold weather and smoke exposure, as well as varying levels of understanding about asthma causes and management [49]. Misconceptions about disease transmission were also reported among some participants. These insights suggest that health literacy and environmental exposures may influence asthma control in ways not captured by the quantitative variables examined in this study.

Among participants with diabetes, no demographic or clinical characteristics were significantly associated with glycaemic control. Although individuals receiving monotherapy demonstrated higher rates of control compared with those receiving multiple medications, this association likely reflects differences in disease severity rather than a causal relationship between medication number and control status [8,32,33,39,50]. Patients requiring multiple medications may have more advanced disease or more complex metabolic profiles, which could reduce the likelihood of achieving glycaemic targets despite appropriate treatment [51,52]. Similar severity-related confounding may explain the observed association between the number of antihypertensive medications and blood pressure control. Patients receiving more intensive pharmacotherapy are often those with treatment-resistant hypertension or more severe disease, which may make achieving optimal control more challenging [42,43,53]. These interpretations are very important when considering underlying disease severity when interpreting associations between treatment intensity and disease outcomes.

Integration of qualitative and quantitative findings suggests that behavioural and health system factors may play an important role in disease management. Participants frequently emphasised medication adherence, dietary modification, and avoidance of environmental triggers as key strategies for managing their conditions. At the same time, logistical barriers such as transportation costs and long travel distances to health facilities were commonly reported. These qualitative insights help explain why demographic characteristics alone were not strongly associated with disease control in the quantitative analysis. Instead, disease management may depend more heavily on patient knowledge, self-care behaviours, and access to consistent follow-up services [10,13,24,31,32,34,51,52]. This mixed-methods perspective highlights the complex interplay between clinical treatment, patient behaviour, and health system accessibility in chronic disease management.

Several limitations should be considered when interpreting these findings. First, the cross-sectional design limits the ability to establish causal relationships between the observed variables and disease control outcomes. Associations identified in the analysis may reflect unmeasured confounding factors, including disease severity, healthcare utilisation patterns, or socio-economic influences. Secondly, disease control status was determined using the most recent clinical measurement recorded in the electronic medical record. This approach may misclassify patients with fluctuating or poorly documented disease trajectories and does not capture sustained disease control over time.

Thirdly, asthma control was assessed using symptom-based indicators rather than objective measures such as spirometry, which may introduce reporting bias and affect comparability with other studies. Fourthly, the relatively small sample sizes for certain subgroups, particularly diabetes, limit statistical power and may reduce the ability to detect meaningful associations.

Fifth, blood pressure measurements were obtained from routine clinical records, and information on cuff size standardisation was not consistently documented. If appropriately sized cuffs were not used for individuals with higher body mass index, this may have introduced systematic measurement error, potentially overestimating blood pressure values among overweight or obese participants and biasing the observed association between BMI and hypertension control.

Sixth, for diabetes and asthma, the analyses of factors associated with disease control were limited to bivariate methods and did not adjust for potential confounders using multivariable models. As a result, the probability of residual confounding is considerable, and the observed associations for these conditions should be interpreted with caution.

Finally, because the study population consisted of patients already engaged in clinic-based care, the results may be influenced by survivorship or care-seeking bias and may not reflect the broader population of individuals living with NCDs in the community.

Despite these limitations, the study provides insights into factors associated with disease control among patients receiving integrated NCD care in a rural setting. Rather than demonstrating the effectiveness of a specific care model, the findings highlight areas that may warrant attention in efforts to strengthen chronic disease management. These include improving patient education regarding disease mechanisms and triggers, addressing logistical barriers to consistent follow-up, and supporting adherence to lifestyle and medication recommendations. Future research using longitudinal designs and comparative analyses across different models of care would be valuable for evaluating how integrated chronic care programmes influence long-term NCD outcomes in resource-limited settings.
