# Supplementary material for: Disease control and its correlates among hypertensive, diabetic, and asthmatic patients enrolled in secondary-level Integrated Chronic Care Clinics in Neno District, rural Malawi
Source: PLOS Glob Public Health. 2026 Jul 30;6(7):e0006629. doi: 10.1371/journal.pgph.0006629 (PMC13423058; doi:10.1371/journal.pgph.0006629)
Supplement: Attachment — Submitted filename: IC3 control NCDs Neno - PGPH review 19May2025.docx [file pgph.0006629.s001.docx]

**Assessing Factors Associated with Disease Control among Hypertensive, Diabetic and Asthmatic Patients Enrolled in Secondary-Level Integrated Chronic Care Clinics in Neno District, Rural Malawi**

GD Limwado et al

PGPH-D-25-00866

Review: J van Oosterhout

Date 19 May 2025

This is a retrospective cohort or cross-sectional and mixed methods study in Neno district in rural Malawi, where with funding from Partners in Health, integrated chronic care clinics have been established for HIV and NCD management. The authors wanted to explore factors associated with diabetes, hypertension and asthma disease control. The manuscript is well written but has a number of limitations. Several points need to be addressed before I would recommend acceptance

***Major points***

1 The Methodology

It is not easy to understand how participants were sampled for each of the 2 components of the study. Were all individuals in care at the 2 HFs with the 3 conditions included for the quantitative survey? How were the sample size and the selection of the participants for the interviews determined?

The authors say that they conducted a retrospective cohort study, but the results suggest that this is a cross-sectional study.

The study seems to have included adults only, were there other exclusion criteria? Were type 1 DM patients included?

Part of the qualitative methodology comes back in the Results and may be moved to the Methods. Definitions of disease control do not belong in the Introduction and may be moved to the Methods. How are disease control outcomes exactly defined? Were BP, HbA1C and asthma symptoms determined cross sectionally at enrolment or retrospectively, i.e. reviewed over several points earlier in time? If the latter, how was control defined? If the former, what are the limitations of such a one-off approach?

The FGD numbers conducted seem unbalanced by disease category.

To improve the Methods section, the authors may consider describing the quantitative and qualitative study components with their methodology separately

2 Lack of a robust statistical analysis

How was the logistic regression analysis performed? A detailed description is needed in the results. Was a multivariable analysis done? If so, how were models constructed? If not, why was this omitted? Without statistical adjustment, the probability of confounded associations is high, strongly limiting the interpretation of their main results. This is acknowledged but it is not explained why multivariable LR could not be done and what that means for the interpretation at the end where “large” conclusions are drawn

The authors say that: “The study carries a high risk of bias due to its reliance on pre-existing data, which may include incomplete or inaccurate information”. While this is a common issue with routinely collected data, readers do not get insight into the degree of completeness and reliability of the data that are presented in this study, which is needed to have any confidence in the results at all

3 Gaps in Results and consequences for the Conclusion

The conclusions of the study “This study underscores the effectiveness of integrated care models like IC3 in managing NCDs in resource-limited settings” does not seem to be well-supported by the data of this study. The levels of overall disease control are not indicated in the text, nor clearly in the Table 2, where we can see control by characteristics, but not overall. Therefore, readers have no easy way of deciding if the IC3 model is effective in achieving adequate disease control. The factors associated with control are outlined in the text but only with p-values, lacking point estimates, which need to be provided. If I am correct, I could determine from Table 2 that overall DM control was around 33% and hypertension control around 55%: do those rates underline success of the IC3 model?

4 Focus of the Discussion

The Discussion is somewhat superficial, providing several points that are long- and well-established facts (e.g. inhaled steroids are beneficial for asthma control), but it does not convince to “underscore the importance of strengthening clinical care practices within the IC3 model”. In the Discussion, can the authors do more to prioritize the main aim of the study and the reason why they said they did the study: “specific factors within the integrated care approach that drive these positive outcomes have not yet been fully identified, leaving opportunities for targeted improvements and reinforcement within this model of care”

***Minor points***

I suggest that throughout the manuscript, authors should use the term significant only in the statistical sense, and not to indicate that an item is substantial or large (“A significant proportion (n=391, 87%)”)

The title indicates that IC3 was studied at secondary level, which may limit representativeness and should be considered as a limitation

Are the disease prevalence rates in the Abstract (and Introduction) from the general population or for specific (age) groups? This should be clarified
