# Supplementary material for: Disease control and its correlates among hypertensive, diabetic, and asthmatic patients enrolled in secondary-level Integrated Chronic Care Clinics in Neno District, rural Malawi
Source: PLOS Glob Public Health. 2026 Jul 30;6(7):e0006629. doi: 10.1371/journal.pgph.0006629 (PMC13423058; doi:10.1371/journal.pgph.0006629)
Supplement: Attachment — Submitted filename: Respsone to reviewers I.docx [file pgph.0006629.s003.docx]

30^th^ August 2025

Helen Howard

Staff Editor

PLOS Global Public Health

Dear Helen,

Thank you kindly for your review of our manuscript *“Assessing Factors Associated with Disease Control among Hypertensive, Diabetic and Asthmatic Patients Enrolled in Secondary-Level Integrated Chronic Care Clinics in Neno District, Rural Malawi”* (PGPH-D-25-00866). The editorial and reviewers’ feedback have significantly strengthened this work. Below, we have offered point-by-point responses to each critique. Please do not hesitate to reach out with further queries.

Warm Regards,

Dr. George Dalitso Limwado, **MBBS**, **MBA-HA**

Senior Medical Officer

Partners in Health/ Abwenzi Pa Za Umoyo

**Journal Requirements**:

1. *Please ensure that your Ethics Statement is available in its entirety at the beginning of your Methods section, under a subheading 'Ethics Statement'.*

Thank you for highlighting this. We have moved the Ethics statement to the beginning of the Methods section.

2. *Tables should not be uploaded as individual files. Please remove these files and include the Tables in your manuscript file as editable, cell-based objects. For more information about how to format tables, see our guidelines:* [*https://journals.plos.org/globalpublichealth/s/tables*](https://journals.plos.org/globalpublichealth/s/tables)

Thank you for mentioning this recommendation. The tables have been removed as separate files and placed into the manuscript.

3. *We note that “Support Letter 1.pdf” and “Support Letter 2.pdf” should not be published with the paper and are for internal information only. We have changed the file type to 'Other' on your behalf to ensure that they are not published.*

We would like to thank the editorial team for changing this on our behalf.

4. *In the online submission form, you indicated that “Data will be provided upon request”. All PLOS journals now require all data underlying the findings described in their manuscript to be freely available to other researchers, either
   - In a public repository, 
   - Within the manuscript itself, or 
   - Uploaded as supplementary information.*

*This policy applies to all data except where public deposition would breach compliance with the protocol approved by your research ethics board. If your data cannot be made publicly available for ethical or legal reasons (e.g., public availability would compromise patient privacy), please explain your reasons by return email and your exemption request will be escalated to the editor for approval. Your exemption request will be handled independently and will not hold up the peer review process, but it will need to be resolved should your manuscript be accepted for publication. One of the Editorial team will then be in touch if there are any issues.*

*If the reviewer comments include a recommendation to cite specific previously published works, please review and evaluate these publications to determine whether they are relevant and should be cited. There is no requirement to cite these works unless the editor has indicated otherwise.* 

We apologise for this. We have sent an exemption request as guided.

**Reviewers' comments**:

5. *It is not easy to understand how participants were sampled for each of the 2 components of the study. Were all individuals in care at the 2 HFs with the 3 conditions included for the quantitative survey?*

We apologise for not clarifying this. We recruited all adults (18 years or above) who were active in care. For the qualitative part, we randomly sampled recipients of care who were present on the day of the interviews.

6. *How were the sample size and the selection of the participants for the interviews determined?*

We sampled all patients who met the criteria. For the interviews, participants were randomly selected on the day of the interview. Following qualitative research principles, we interviewed participants until we reached saturation of responses.

7. *The authors say that they conducted a retrospective cohort study, but the results suggest that this is a cross-sectional study.*

We apologise for the oversight, though the data was from a cohort of patients, the analysis was done cross-sectionally. Therefore, the study description has been revised.

8. *The study seems to have included adults only; were there other exclusion criteria? Were type 1 DM patients included?*

This study focused on recipients of care from our integrated chronic care clinic (WHO-Pen), which does not have DM type 1 patients. These are seen in our advanced NCD clinic (WHO – Pen+)

9. *Part of the qualitative methodology comes back in the Results and may be moved to the Methods. Definitions of disease control do not belong in the Introduction and may be moved to the Methods. How are disease control outcomes exactly defined? Were BP, HbA1C and asthma symptoms determined cross-sectionally at enrolment or retrospectively, i.e. reviewed over several points earlier in time? If the latter, how was control defined? If the former, what are the limitations of such a one-off approach?*

We would like to thank the reviewers for highlighting this. Part of the qualitative methodology has been moved to the methods section, definition of diseases has been moved to the methods section as well.

Disease control was determined cross-sectionally, which has the inability to determine causal relationships or track disease control dynamics over time.

10. *The FGD numbers conducted seem unbalanced by disease category.*

We apologise for this oversight. The FGD numbers have been balanced

11. *To improve the Methods section, the authors may consider describing the quantitative and qualitative study components with their methodology separately*

We are very grateful for this suggestion; it has been considered and applied.

12. *How was the logistic regression analysis performed? A detailed description is needed in the results. Was a multivariable analysis done? If so, how were models constructed? If not, why was this omitted? Without statistical adjustment, the probability of confounded associations is high, strongly limiting the interpretation of their main results. This is acknowledged, but it is not explained why multivariable LR could not be done and what that means for the interpretation at the end, where “large” conclusions are drawn.*

No logistic regression was done; this statement has been removed from the manuscript. The Fisher exact test was done instead.

13. *The authors say that: “The study carries a high risk of bias due to its reliance on pre-existing data, which may include incomplete or inaccurate information”. While this is a common issue with routinely collected data, readers do not get insight into the degree of completeness and reliability of the data that are presented in this study, which is needed to have any confidence in the results at all.*

We appreciate the reviewer’s point. We agree that stating “high risk of bias” without providing context may limit the reader’s ability to judge the reliability of our findings. In response, we have added detail on the completeness and reliability of our dataset, including the extent of missingness and the data quality assurance procedures that were in place during data collection.

14. *The conclusions of the study, “This study underscores the effectiveness of integrated care models like IC3 in managing NCDs in resource-limited settings” do not seem to be well-supported by the data of this study. The levels of overall disease control are not indicated in the text, nor clearly in Table 2, where we can see control by characteristics, but not overall. Therefore, readers have no easy way of deciding if the IC3 model is effective in achieving adequate disease control. The factors associated with control are outlined in the text, but only with p-values, lacking point estimates, which need to be provided.*

The statement will be removed. And point estimates have been provided.

15. *If I am correct, I could determine from Table 2 that overall DM control was around 33% and hypertension control around 55%: do those rates underline the success of the IC3 model?*

No, this does not underline the success of the IC3 model. Revisions have been made where such generalisations were made.

16. *The Discussion is somewhat superficial, providing several points that are long and well-established facts (e.g. inhaled steroids are beneficial for asthma control), but it does not convince to “underscore the importance of strengthening clinical care practices within the IC3 model”.*

We agree with the reviewer; therefore, the whole discussion section has been revised. The revised version has focused on the study’s main aim.

17. *In the Discussion, can the authors do more to prioritize the main aim of the study and the reason why they said they did the study: “specific factors within the integrated care approach that drive these positive outcomes have not yet been fully identified, leaving opportunities for targeted improvements and reinforcement within this model of care.”*

Thank you for the guidance. We have revised the discussion section and focused on the aim of the study.

18. *I suggest that throughout the manuscript, authors should use the term significant only in the statistical sense, and not to indicate that an item is substantial or large (“A significant proportion (n=391, 87%)”)*

These have been replaced, and all sections with this have been revised.

19. *The title indicates that IC3 was studied at the secondary level, which may limit representativeness and should be considered as a limitation.*

This is noted and acknowledged as a limitation.

20. *Are the disease prevalence rates in the Abstract (and Introduction) from the general population or for specific (age) groups? This should be clarified.*

Thank you for flagging this out. We have revised the abstract and clarified as directed.

21. *The text of the article mentions the WHO PEN protocol, but the entire study is not consistent with the integrated care of non-communicable diseases such as diabetes and hypertension.*

We acknowledge the reviewer’s view and we have revised multiple sections of the article to reflect how the IC3 model follows the WHO Pen protocols.

22. *The lack of assessment of 10-year cardiovascular risk is also clearly evident in the article.*

We acknowledge this, and we have highlighted it as one of our limitations.

23. *At first, I thought that access to cholesterol testing was limited, but when it is possible to measure glycosylated haemoglobin (HbA1c), for example, lipid profile assessment is also possible.*

This is true, the truth of the matter is that the HbA1c are donated, that is why we are able to run that test, but not cholesterol testing.

24. *Also, the reason for removing COPD is not known because it is one of the components of the PEN protocol.*

We acknowledge this omission; COPD has been added, though it is a cohort of 24 recipients of care without any significance as far as factors associated with disease control are concerned.

25. *I have a question that how overweight is mentioned in the case of diabetics. Were none of the patients obese? Was the body mass index of all of them less than 30?*

We acknowledge this, and yes, some patients were indeed obese. We categorised that variable into 3 categories, i.e. underweight, normal and overweight; that is why obese does not appear in the manuscript.
