# Supplementary material for: Disease control and its correlates among hypertensive, diabetic, and asthmatic patients enrolled in secondary-level Integrated Chronic Care Clinics in Neno District, rural Malawi
Source: PLOS Glob Public Health. 2026 Jul 30;6(7):e0006629. doi: 10.1371/journal.pgph.0006629 (PMC13423058; doi:10.1371/journal.pgph.0006629)
Supplement: Attachment — Submitted filename: Response to reviwers 2.docx [file pgph.0006629.s006.docx]

31^st^ October 2025

Jianhong Zhou
Staff Editor
PLOS Global Public Health

Dear Jianhong,

Thank you kindly for your review of our manuscript **“Assessing Factors Associated with Disease Control among Hypertensive, Diabetic and Asthmatic Patients Enrolled in Secondary-Level Integrated Chronic Care Clinics in Neno District, Rural Malawi” (PGPH-D-25-00866)**. The editorial and reviewers’ feedback have significantly strengthened this work. Below, we have offered point-by-point responses to each critique. Please do not hesitate to reach out with further queries.

Warm Regards,

Dr. George Dalitso Limwado, MBBS, MBA-HA

Senior Medical Officer

Partners in Health/ Abwenzi Pa Za Umoyo

**Journal Requirements**:

1. If the reviewer comments include a recommendation to cite specific previously published works, please review and evaluate these publications to determine whether they are relevant and should be cited. There is no requirement to cite these works unless the editor has indicated otherwise.

- Thank you for highlighting that; the reviewers did not comment about our cited published works.

**Reviewers’ comments**

1. Some info is included in the response to comments but not in the R1 text: the exclusion of individuals with type 1 DM is an example, and another one is how disease control was determined (one-off result at the last measurement instead of involving multiple results). This needs to be rectified.

- We apologize for the oversight and we thank the reviewer for highlighting this. We have now clarified in the manuscript that individuals with type 1 diabetes mellitus were excluded because, within the Neno Integrated Chronic Care Clinic (IC3) model, patients requiring insulin (classified as type 1 diabetes or advanced disease) are followed in the Advanced NCD Clinic, not in the IC3 cohort assessed in this study.

We have also clarified how disease control was determined. Specifically, control was assessed using a one-off measurement at the most recent clinic visit, rather than through longitudinal data, because many recipients of care lacked complete serial measurements. We have acknowledged this as a methodological limitation and acknowledged it in the discussion as a limitation. Being cross sectional study, we took the last test result.

2. Different, higher numbers have appeared in the results of the quantitative analysis in the R1 document – it is not clear to me why. It is not explained by adding COPD patients at the request of Reviewer 2. There are now large discrepancies between the numbers in the abstract and the results.

- Thank you for this observation. The discrepancy arose following a comprehensive data validation step conducted after Reviewer 2’s suggestion to include COPD cases. During this process, we corrected disease group classifications and updated the counts to reflect the complete, cleaned dataset extracted from OpenMRS. The total number of unique recipients of care (ROCs) enrolled in the study remained 1,152, consistent with our initial report. However, individual disease categories (hypertension, diabetes, asthma, COPD) were recalculated to reflect more accurate counts based on verified diagnostic coding and removal of duplicates across comorbid conditions.
- The updated stratification is as follows:
- Hypertension: 1,301 cases (including those with comorbidities)
- Asthma: 451 cases
- Diabetes mellitus: 87 cases
- COPD: 24 cases
- These represent diagnostic categories, not mutually exclusive groups. The figures in the revised abstract and results now align with the cleaned dataset, providing a more accurate representation of disease distribution within the 1,152 unique ROCs.

3. In response to the question: “The FGD numbers conducted seem unbalanced by disease category”, the authors say that the numbers have now been balanced, but how can that be done after the study was completed, as the completed FGDs determined these numbers. Or have I misunderstood something?

- Put it as a limitation. We appreciate the reviewer’s observation. We would like to clarify that no additional focus group discussions (FGDs) or interviews were conducted after the completion of data collection. The term “balanced” in our previous response may have been misleading. What we meant was that, during the data analysis and manuscript revision process, we re-verified and clarified the distribution of participants across disease categories to ensure that each condition (hypertension, diabetes, and asthma) was adequately represented in the qualitative data.

The apparent imbalance noted earlier was due to reporting inconsistencies rather than new data collection. Upon review, we confirmed that a total of 17 FGDs and 56 in-depth interviews were indeed conducted as originally planned. Within these, participants represented all three disease categories—14 with hypertension, 13 with asthma, and 13 with diabetes—along with one participant presenting both hypertension and asthma. These distributions reflect the natural clinic attendance patterns within the IC3 model rather than any post-study adjustments.

4. The authors indicate that a multivariable analysis was not done, but they do explain why. It leaves the analysis of factors associated with disease control open to a high probability of confounding bias, which cannot be addressed by a low degree of data “missingness and the data quality assurance procedures in place (response of the authors).

- We acknowledge that the absence of a multivariable analysis in the initial submission limited our ability to adjust for potential confounders. In response, we have now conducted a multivariable analysis to account for these confounding factors. The results and corresponding interpretations have been incorporated into the Results section, and the Discussion section has been updated accordingly to reflect these findings.
