# Supplementary material for: Disease control and its correlates among hypertensive, diabetic, and asthmatic patients enrolled in secondary-level Integrated Chronic Care Clinics in Neno District, rural Malawi
Source: PLOS Glob Public Health. 2026 Jul 30;6(7):e0006629. doi: 10.1371/journal.pgph.0006629 (PMC13423058; doi:10.1371/journal.pgph.0006629)
Supplement: Attachment — Submitted filename: PGPH-D-25-00866.R2 Review for authors.docx [file pgph.0006629.s004.docx]

**Assessing Factors Associated with Disease Control among Hypertensive, Diabetic and Asthmatic Patients Enrolled in Secondary-Level Integrated Chronic Care Clinics in Neno District, Rural Malawi**

GD Limwado et al

PGPH-D-25-00866.R2

**Major issues**

1. Discrepancies in the number of participants

The numbers are still different between sections of the manuscript and in the response to reviewers. In the abstract we read: *“Of the 1,152 ROCs, hypertension was the most prevalent condition (n=1,301 cases)……”*. In the Methods we read: *“A total of 1,152 patients were included, categorised by diagnosis as follows: 956 with hypertension, 56 with type 2 diabetes, 24 with COPD and 116 with asthma”*, while in the response to reviewer (point 2) it is stated that: *“The total number of unique recipients of care (ROCs) enrolled in the study remained 1,152, consistent with our initial report. However, individual disease categories (hypertension, diabetes, asthma, COPD) were recalculated to reflect more accurate counts based on verified diagnostic coding and removal of duplicates across comorbid conditions. The updated stratification is as follows: Hypertension: 1,301 cases (including those with comorbidities)……”*. How is it possible to enroll 1,152 ROCs and have 1,301 cases with hypertension or were there 956 cases with hypertension? These discrepancies continue (this was pointed out in previous reviewer comments) to cause confusion and question reliability.

2. The addition of COPD patients

In the Methods (Inclusion criteria), we read: *“Participants were required to be 18 years or older, diagnosed with diabetes, hypertension, or asthma, and receiving care through IC3 between January 2017 and December 2021”*. But in the response to reviewer, we understand that a group with COPD was included – which was overlooked in the Methods. In addition, control of COPD is not defined in the Methods. It appears the authors have just added a column of COPD patients without attention to the required details. Furthermore, I doubt the meaningfulness of adding a very small number of COPD patients in the analysis

3. The multivariable analysis

The authors have conducted a multivariable analysis, but this is not mentioned at all in the Methods, which was left unchanged. The table with results of a multivariable analysis in the results is not easy to oversee and difficult to understand, as the methodology is missing. Under this Table 3, we read: *“For asthma, the odds of disease control were lower among males compared to females (aOR = 0.59, 95% CI 0.33–1.10; p = 0.096)”*, but the confidence interval includes 1 and the p-value indicates a non-significant association.

How is it possible that male sex was significantly negatively associated with controlled hypertension, aOR 0.70 (0.55 - 0.90), while a larger percentage of men (57%) had controlled hypertension than women (49%)? This is remarkable, though possible, however it seems that the authors did not notice nor discussed it. In Table 3, I note that the aOR of being on 2 drugs (vs. 1 drug as reference) for asthma control is 0.11 (0.05 - 6.48), p-value <0.001. How is it possible that if the confidence interval includes 1, the p-value can indicate strong statistical significance? I assume this is a mistake. In addition, in table 2, we see that 96% of patients on 1 asthma drug had asthma control vs. 76% of patients on two drugs! In the Discussion the authors argue *“use of combined inhaled steroids and beta-agonist inhalers* (i.e. being on 2 drugs) *was strongly associated with asthma control”* and *“…underscores IC3’s success in facilitating access to evidence-based asthma management in a rural, resource-limited context”,* but the aOR of asthma control when being on 2 drugs was 0.11.

4. Findings and conclusions

The authors say in the Discussion that: *“Notably, clients on monotherapy demonstrated better glycaemic control than those on dual therapy (p = 0.031), a finding that likely indicates less severe disease but also suggests that early detection and consistent follow-up, both facilitated by IC3’s community outreach, support more effective disease control”*, yet in the multivariable analysis, the number of drugs used was not associated with diabetes control. Hos should this be interpreted?

All the conclusions about associations with control of asthma, COPD and diabetes need to be watered down strongly as the numbers are very small, limiting the power to detect associations and reducing the meaning of the results. Still the authors come up with narrative such as: *“Our findings provide critical insights that can be used to strengthen clinical practice, inform targeted improvements, and guide the implementation of similar models in other settings”*

**Minor issues**

- NCD control prevalence ratios need to be provided in the abstract
- The study period is different in the Abstract and the Methods
- Inclusion criteria for the qualitative component are mentioned in the paragraph describing the quantitative component
- Table titles appear below the tables, instead of above
- In the Results section below Table 3, we read there that: *“…..having one comorbid condition (aOR = 3.92, 95% CI 1.08–14.22; p = 0.038) was significantly associated with better asthma control compared to having none, suggesting that individuals already managing another condition may be more engaged with healthcare services”*. This reason for the observed association seems speculative. In addition, this text should not appear in the Results section but in the Discussion.
- In the Results of the qualitative study, first paragraph, parts of the methodology are described. Such narrative should appear in the Methods section
