# Supplementary material for: Disease control and its correlates among hypertensive, diabetic, and asthmatic patients enrolled in secondary-level Integrated Chronic Care Clinics in Neno District, rural Malawi
Source: PLOS Glob Public Health. 2026 Jul 30;6(7):e0006629. doi: 10.1371/journal.pgph.0006629 (PMC13423058; doi:10.1371/journal.pgph.0006629)
Supplement: Attachment — Submitted filename: Reviewer Comments.docx [file pgph.0006629.s005.docx]

**ASSESSING FACTORS ASSOCIATED WITH DISEASE CONTROL AMONG HYPERTENSIVE, DIABETIC, AND ASTHMATIC PATIENTS ENROLLED IN SECONDARY-LEVEL INTEGRATED CHRONIC CARE CLINICS IN NENO DISTRICT, RURAL MALAWI**

**Reviewer Comments**

**Title and Abstract**

The title implies a comprehensive assessment of factors associated with disease control, yet the quantitative analyses identify very few robust predictors, especially after multivariable adjustment. This creates a mismatch between the title and actual inferential strength of the results.

The abstract overstates conclusions regarding the “effectiveness” of the IC3 model, despite the absence of a comparator group or pre-post analysis to support effectiveness claims.

**Introduction**

While well-referenced, the introduction lacks a clear conceptual or analytical framework explaining how integrated care is hypothesized to influence disease control outcomes.

The research gap is framed broadly; however, the specific hypotheses tested in the quantitative analysis are not clearly articulated, making it difficult to assess whether the analyses are confirmatory or exploratory.

**Methods**

**Study Design and Population**

The retrospective cross-sectional design does not allow causal inference, yet the manuscript repeatedly implies causal relationships (e.g., medication number influencing control).

There are internal inconsistencies in dates (e.g., February-December 2022 vs July-December 2023 vs 2017-2021 enrollment), which raise concerns about data coherence and require clarification.

**Disease Definitions and Measurement**

Disease control is based on single most recent measurements, which may misclassify chronic control status and introduces measurement bias. This limitation is acknowledged but not sufficiently accounted for in interpretation.

Asthma control relies on self-reported symptoms without objective measures, increasing risk of reporting bias, particularly in qualitative-quantitative integration.

**Sample Size and Power**

Subgroup sizes for diabetes and COPD are very small, yet multivariable regression is performed. This raises serious concerns regarding model overfitting, unstable estimates, and wide confidence intervals.

**Statistical Analysis**

Variable selection for multivariable models is not justified (e.g., no explanation of inclusion criteria or handling of collinearity).

The number of predictors relative to outcome events, especially for diabetes and asthma models, violates recommended events-per-variable thresholds, undermining the reliability of adjusted odds ratios.

Several statistically significant univariate associations disappear in multivariable models, but this discordance is not critically discussed.

**Results**

Tables are dense and difficult to interpret, with limited narrative synthesis highlighting clinically meaningful findings.

The finding that “fewer medications” is associated with better control is likely confounded by disease severity, yet this alternative explanation is insufficiently explored.

COPD results are reported despite an extremely small sample (n=24), limiting interpretability and adding little value to the analysis.

**Qualitative Component**

While rich in quotations, the qualitative analysis lacks methodological depth:

No reflexivity statements

Limited description of coder training or intercoder reliability

Integration of qualitative and quantitative findings is largely descriptive, rather than analytically integrated to explain discordant or null quantitative results.

**Discussion**

The discussion overinterprets associations as indicators of IC3 effectiveness without appropriate comparative data.

Alternative explanations (e.g., survivorship bias, care-seeking behavior bias, severity confounding) are not adequately explored.

The policy implications are strong, but the evidence base presented does not fully support scaling recommendations.

**Limitations**

Although limitations are listed, they are not sufficiently weighted in the conclusions. Key methodological constraints, particularly cross-sectional design, measurement limitations, and small subgroup sizes, should more explicitly temper claims.

**Data Availability and Ethics**

The data availability statement imposes multiple access barriers. While ethically justified, the authors should clarify how this aligns with PLOS data-sharing expectations and whether metadata or analytic code can be shared.
