# Supplementary material for: Disease control and its correlates among hypertensive, diabetic, and asthmatic patients enrolled in secondary-level Integrated Chronic Care Clinics in Neno District, rural Malawi
Source: PLOS Glob Public Health. 2026 Jul 30;6(7):e0006629. doi: 10.1371/journal.pgph.0006629 (PMC13423058; doi:10.1371/journal.pgph.0006629)
Supplement: Attachment — Submitted filename: PGPH-D-25-00866.R3 Review JvO.docx [file pgph.0006629.s007.docx]

**Assessing Factors Associated with Disease Control among Hypertensive, Diabetic and Asthmatic Patients Enrolled in Secondary-Level Integrated Chronic Care Clinics in Neno District, Rural Malawi**

GD Limwado et al

PGPH-D-25-00866.R3

Review:	J van Oosterhout

Date:	22 March 2026

Most comments have been addressed and the manuscript is now nearly ready for publication in my view

I still have a number of minor points for improvement:

General: use the same number of decimals for percentages consistently throughout all sections of the manuscript

Abstract (Methods): describe which associations in: *Associations…..logistic regression.*

Methods: was the cuff size adapted to BMI, in particular, were appropriate cuffs used of obese and overweight individuals? This may have repercussions for the association of BMI with BP control. If the same cuff size was used for all, I suggest that this needs to be added to the limitations

Methods (Sample size): this sentence is a Result: *“A total of…..with Asthma.”* I suggest placing it in the Results and add a description of the overlap of conditions among participants.

Methods (Participant recruitment): I suggest adding information about how the randomization was conducted.

Methods (Statistical analysis): please add some details on the methodology of the multivariable logistic regression analysis, for instance, was backward elimination used or otherwise?

Results (bivariate analysis): the point estimates with CI (not just the p-values) need to be provided with significant associations, also in the abstract

Results: each of the three following paragraphs are interpretations of results and should appear in the Discussion in my view:

*“These mixed levels of health literacy help explain the quantitative findings, which showed no consistent association between demographic factors and disease control for asthma and diabetes. Instead, variation in knowledge and beliefs about disease mechanisms may influence adherence and symptom management beyond measured clinical variables.”*

*“Such barriers may contribute to delayed care-seeking or irregular follow-up. These accounts provide context for the quantitative finding that a substantial proportion of participants had uncontrolled disease, particularly among individuals with diabetes and hypertension. Travel burden and financial constraints may limit continuity of care, medication refills, and monitoring visits necessary for optimal disease control.”*

*“These narratives align with the quantitative findings showing associations between medication regimens and disease control, particularly for hypertension. Effective communication and counselling appear central to supporting adherence to both medication and lifestyle modifications.”*

Discussion: please add to the limitations section that the probability of confounding is considerable in the analyses of factors associated with diabetes and asthma control, as the analyses did not correct for confounding statistically.
