# Supplementary material for: Disease control and its correlates among hypertensive, diabetic, and asthmatic patients enrolled in secondary-level Integrated Chronic Care Clinics in Neno District, rural Malawi
Source: PLOS Glob Public Health. 2026 Jul 30;6(7):e0006629. doi: 10.1371/journal.pgph.0006629 (PMC13423058; doi:10.1371/journal.pgph.0006629)
Supplement: Peer review history

# Peer review: Decision Letter 0

**DOI:** 10.1371/journal.pgph.0006629.r001

**Authors:** Helen Howard

18 Jul 2025

PGPH-D-25-00866

Assessing Factors Associated with Disease Control among Hypertensive, Diabetic and Asthmatic Patients Enrolled in Secondary-Level Integrated Chronic Care Clinics in Neno District, Rural Malawi.

PLOS Global Public Health

Dear Dr. Limwado,

Thank you for submitting your manuscript to PLOS Global Public Health. After careful consideration, we feel that it has merit but does not fully meet PLOS Global Public Health’s publication criteria as it currently stands. Therefore, we invite you to submit a revised version of the manuscript that addresses the points raised during the review process.

The manuscript has been evaluated by two reviewers, and their comments are available below.

The reviewers have raised a number of concerns that need attention. In particular, they request additional information on the methodological and statistical aspects of the study, and revisions to the discussion and conclusions sections.

Could you please revise the manuscript to carefully address the concerns raised?

Please submit your revised manuscript by Aug 31 2025 11:59PM. If you will need more time than this to complete your revisions, please reply to this message or contact the journal office at globalpubhealth@plos.org. When you're ready to submit your revision, log on to https://www.editorialmanager.com/pgph/ and select the 'Submissions Needing Revision' folder to locate your manuscript file.

Please include the following items when submitting your revised manuscript:

- A rebuttal letter that responds to each point raised by the editor and reviewer(s). You should upload this letter as a separate file labeled 'Response to Reviewers'.
- A marked-up copy of your manuscript that highlights changes made to the original version. You should upload this as a separate file labeled 'Revised Manuscript with Track Changes'.
- An unmarked version of your revised paper without tracked changes. You should upload this as a separate file labeled 'Manuscript'.

Guidelines for resubmitting your figure files are available below the reviewer comments at the end of this letter.

We look forward to receiving your revised manuscript.

Kind regards,

Helen Howard

Staff Editor

PLOS Global Public Health

Journal Requirements:

1. Please ensure that your Ethics Statement is available in its entirety at the beginning of your Methods section, under a subheading 'Ethics Statement'.

2. Tables should not be uploaded as individual files. Please remove these files and include the Tables in your manuscript file as editable, cell-based objects. For more information about how to format tables, see our guidelines:

https://journals.plos.org/globalpublichealth/s/tables

3. We note that “Support Letter 1.pdf” and “Support Letter 2.pdf” should not be published with the paper and are for internal information only. We have changed the file type to 'Other' on your behalf to ensure that they are not published.

4. In the online submission form, you indicated that “Data will be provided upon request”.

All PLOS journals now require all data underlying the findings described in their manuscript to be freely available to other researchers, either

1. In a public repository,

2. Within the manuscript itself, or

3. Uploaded as supplementary information.

This policy applies to all data except where public deposition would breach compliance with the protocol approved by your research ethics board. If your data cannot be made publicly available for ethical or legal reasons (e.g., public availability would compromise patient privacy), please explain your reasons by return email and your exemption request will be escalated to the editor for approval. Your exemption request will be handled independently and will not hold up the peer review process, but will need to be resolved should your manuscript be accepted for publication. One of the Editorial team will then be in touch if there are any issues.

If the reviewer comments include a recommendation to cite specific previously published works, please review and evaluate these publications to determine whether they are relevant and should be cited. There is no requirement to cite these works unless the editor has indicated otherwise.

Additional Editor Comments (if provided):

[Note: HTML markup is below. Please do not edit.]

Reviewers' comments:

Reviewer's Responses to Questions

**Comments to the Author**

1. Does this manuscript meet PLOS Global Public Health’s publication criteria? Is the manuscript technically sound, and do the data support the conclusions? The manuscript must describe methodologically and ethically rigorous research with conclusions that are appropriately drawn based on the data presented.

Reviewer #1: Partly

Reviewer #2: Yes

**********

2. Has the statistical analysis been performed appropriately and rigorously?

Reviewer #1: I don't know

Reviewer #2: Yes

**********

3. Have the authors made all data underlying the findings in their manuscript fully available (please refer to the Data Availability Statement at the start of the manuscript PDF file)?

The PLOS Data policy requires authors to make all data underlying the findings described in their manuscript fully available without restriction, with rare exception. The data should be provided as part of the manuscript or its supporting information, or deposited to a public repository. For example, in addition to summary statistics, the data points behind means, medians and variance measures should be available. If there are restrictions on publicly sharing data—e.g. participant privacy or use of data from a third party—those must be specified.

Reviewer #1: Yes

Reviewer #2: Yes

**********

4. Is the manuscript presented in an intelligible fashion and written in standard English?

PLOS Global Public Health does not copyedit accepted manuscripts, so the language in submitted articles must be clear, correct, and unambiguous. Any typographical or grammatical errors should be corrected at revision, so please note any specific errors here.

Reviewer #1: Yes

Reviewer #2: Yes

**********

5. Review Comments to the Author

Please use the space provided to explain your answers to the questions above. You may also include additional comments for the author, including concerns about dual publication, research ethics, or publication ethics. (Please upload your review as an attachment if it exceeds 20,000 characters)

Reviewer #1: This is a retrospective cohort or cross-sectional and mixed methods study in Neno district in rural Malawi, where with funding from Partners in Health, integrated chronic care clinics have been established for HIV and NCD management. The authors wanted to explore factors associated with diabetes, hypertension and asthma disease control. The manuscript is well written but has a number of limitations.

Major points

1 The Methodology

It is not easy to understand how participants were sampled for each of the 2 components of the study. Were all individuals in care at the 2 HFs with the 3 conditions included for the quantitative survey? How were the sample size and the selection of the participants for the interviews determined?

The authors say that they conducted a retrospective cohort study, but the results suggest that this is a cross-sectional study.

The study seems to have included adults only, were there other exclusion criteria? Were type 1 DM patients included?

Part of the qualitative methodology comes back in the Results and may be moved to the Methods. Definitions of disease control do not belong in the Introduction and may be moved to the Methods. How are disease control outcomes exactly defined? Were BP, HbA1C and asthma symptoms determined cross sectionally at enrolment or retrospectively, i.e. reviewed over several points earlier in time? If the latter, how was control defined? If the former, what are the limitations of such a one-off approach?

The FGD numbers conducted seem unbalanced by disease category.

To improve the Methods section, the authors may consider describing the quantitative and qualitative study components with their methodology separately

2 Lack of a robust statistical analysis

How was the logistic regression analysis performed? A detailed description is needed in the results. Was a multivariable analysis done? If so, how were models constructed? If not, why was this omitted? Without statistical adjustment, the probability of confounded associations is high, strongly limiting the interpretation of their main results. This is acknowledged but it is not explained why multivariable LR could not be done and what that means for the interpretation at the end where “large” conclusions are drawn

The authors say that: “The study carries a high risk of bias due to its reliance on pre-existing data, which may include incomplete or inaccurate information”. While this is a common issue with routinely collected data, readers do not get insight into the degree of completeness and reliability of the data that are presented in this study, which is needed to have any confidence in the results at all

3 Gaps in Results and consequences for the Conclusion

The conclusions of the study “This study underscores the effectiveness of integrated care models like IC3 in managing NCDs in resource-limited settings” does not seem to be well-supported by the data of this study. The levels of overall disease control are not indicated in the text, nor clearly in the Table 2, where we can see control by characteristics, but not overall. Therefore, readers have no easy way of deciding if the IC3 model is effective in achieving adequate disease control. The factors associated with control are outlined in the text but only with p-values, lacking point estimates, which need to be provided.

If I am correct, I could determine from Table 2 that overall DM control was around 33% and hypertension control around 55%: do those rates underline success of the IC3 model?

4 Focus of the Discussion

The Discussion is somewhat superficial, providing several points that are long- and well-established facts (e.g. inhaled steroids are beneficial for asthma control), but it does not convince to “underscore the importance of strengthening clinical care practices within the IC3 model”.

In the Discussion, can the authors do more to prioritize the main aim of the study and the reason why they said they did the study: “specific factors within the integrated care approach that drive these positive outcomes have not yet been fully identified, leaving opportunities for targeted improvements and reinforcement within this model of care”

Minor points

I suggest that throughout the manuscript, authors should use the term significant only in the statistical sense, and not to indicate that an item is substantial or large (“A significant proportion (n=391, 87%)”)

The title indicates that IC3 was studied at secondary level, which may limit representativeness and should be considered as a limitation

Are the disease prevalence rates in the Abstract (and Introduction) from the general population or for specific (age) groups? This should be clarified

Reviewer #2: The text of the article mentions the WHO PEN protocol, but the entire study is not consistent with the integrated care of noncommunicable diseases such as diabetes and hypertension. The lack of assessment of 10-year cardiovascular risk is also clearly evident in the article. At first, I thought that access to cholesterol testing was limited, but when it is possible to measure glycosylated hemoglobin (HbA1c), for example, lipid profile assessment is also possible.

Also, the reason for removing COPD is not known because it is one of the components of the PEN protocol. I have a question that overweight is mentioned in the case of diabetics. Were none of the patients obese? Was the body mass index of all of them less than 30?

**********

6. PLOS authors have the option to publish the peer review history of their article (what does this mean?). If published, this will include your full peer review and any attached files.

**Do you want your identity to be public for this peer review?**  If you choose “no”, your identity will remain anonymous but your review may still be made public.

For information about this choice, including consent withdrawal, please see our Privacy Policy.

Reviewer #1: No

Reviewer #2: No

**********

[NOTE: If reviewer comments were submitted as an attachment file, they will be attached to this email and accessible via the submission site. Please log into your account, locate the manuscript record, and check for the action link "View Attachments". If this link does not appear, there are no attachment files.]

While revising your submission, please upload your figure files to the Preflight Analysis and Conversion Engine (PACE) digital diagnostic tool, https://pacev2.apexcovantage.com/. PACE helps ensure that figures meet PLOS requirements. To use PACE, you must first register as a user. Registration is free. Then, login and navigate to the UPLOAD tab, where you will find detailed instructions on how to use the tool. If you encounter any issues or have any questions when using PACE, please email PLOS at figures@plos.org. Please note that Supporting Information files do not need this step.

Attachment Submitted filename: IC3 control NCDs Neno - PGPH review 19May2025.docx

# Author comment: Author response to Decision Letter 1

**DOI:** 10.1371/journal.pgph.0006629.r002

3 Sep 2025

Attachment Submitted filename: Respsone to reviewers I.docx

# Peer review: Decision Letter 1

**DOI:** 10.1371/journal.pgph.0006629.r003

**Authors:** Jianhong Zhou

17 Sep 2025

PGPH-D-25-00866R1

Assessing Factors Associated with Disease Control among Hypertensive, Diabetic and Asthmatic Patients Enrolled in Secondary-Level Integrated Chronic Care Clinics in Neno District, Rural Malawi.

PLOS Global Public Health

Dear Dr. Limwado,

Thank you for submitting your manuscript to PLOS Global Public Health. After careful consideration, we feel that it has merit but does not fully meet PLOS Global Public Health’s publication criteria as it currently stands. Therefore, we invite you to submit a revised version of the manuscript that addresses the points raised during the review process.

Please address reviewer 1's remaining concerns.

Please submit your revised manuscript by Oct 31 2025 11:59PM. If you will need more time than this to complete your revisions, please reply to this message or contact the journal office at globalpubhealth@plos.org. When you're ready to submit your revision, log on to https://www.editorialmanager.com/pgph/ and select the 'Submissions Needing Revision' folder to locate your manuscript file.

Please include the following items when submitting your revised manuscript:

- A rebuttal letter that responds to each point raised by the editor and reviewer(s). You should upload this letter as a separate file labeled 'Response to Reviewers'.
- A marked-up copy of your manuscript that highlights changes made to the original version. You should upload this as a separate file labeled 'Revised Manuscript with Track Changes'.
- An unmarked version of your revised paper without tracked changes. You should upload this as a separate file labeled 'Manuscript'.

Guidelines for resubmitting your figure files are available below the reviewer comments at the end of this letter.

We look forward to receiving your revised manuscript.

Kind regards,

Jianhong Zhou

Staff Editor

PLOS Global Public Health

Journal Requirements:

If the reviewer comments include a recommendation to cite specific previously published works, please review and evaluate these publications to determine whether they are relevant and should be cited. There is no requirement to cite these works unless the editor has indicated otherwise.

[Note: HTML markup is below. Please do not edit.]

Reviewers' comments:

Reviewer's Responses to Questions

**Comments to the Author**

1. If the authors have adequately addressed your comments raised in a previous round of review and you feel that this manuscript is now acceptable for publication, you may indicate that here to bypass the “Comments to the Author” section, enter your conflict of interest statement in the “Confidential to Editor” section, and submit your "Accept" recommendation.

Reviewer #1: (No Response)

Reviewer #2: All comments have been addressed

**********

2. Does this manuscript meet PLOS Global Public Health’s publication criteria? Is the manuscript technically sound, and do the data support the conclusions? The manuscript must describe methodologically and ethically rigorous research with conclusions that are appropriately drawn based on the data presented.

Reviewer #1: Yes

Reviewer #2: Yes

**********

3. Has the statistical analysis been performed appropriately and rigorously?

Reviewer #1: No

Reviewer #2: Yes

**********

4. Have the authors made all data underlying the findings in their manuscript fully available (please refer to the Data Availability Statement at the start of the manuscript PDF file)?

The PLOS Data policy requires authors to make all data underlying the findings described in their manuscript fully available without restriction, with rare exception. The data should be provided as part of the manuscript or its supporting information, or deposited to a public repository. For example, in addition to summary statistics, the data points behind means, medians and variance measures should be available. If there are restrictions on publicly sharing data—e.g. participant privacy or use of data from a third party—those must be specified.

Reviewer #1: Yes

Reviewer #2: Yes

**********

5. Is the manuscript presented in an intelligible fashion and written in standard English?

PLOS Global Public Health does not copyedit accepted manuscripts, so the language in submitted articles must be clear, correct, and unambiguous. Any typographical or grammatical errors should be corrected at revision, so please note any specific errors here.

Reviewer #1: Yes

Reviewer #2: Yes

**********

6. Review Comments to the Author

Please use the space provided to explain your answers to the questions above. You may also include additional comments for the author, including concerns about dual publication, research ethics, or publication ethics. (Please upload your review as an attachment if it exceeds 20,000 characters)

Reviewer #1: Remaining important issues

1. Some info is included in the response to comments but not in the R1 text: the exclusion of individuals with type 1 DM is an example and another one is how disease control was determined (one-off result at the last measurement instead of involving multiple results). This needs to be rectified

2. Different, higher numbers have appeared in the results of the quantitative analysis in the R1 document – it is not clear to me why. It is not explained by adding COPD patients at the request of Reviewer 2. There are now large discrepancies between the numbers in the abstract and the results.

3. In response to the question: “The FGD numbers conducted seem unbalanced by disease category”, the authors say that the numbers have now been balanced, but how can that be done after the study was completed, as these numbers were determined by the completed FGDs. Or have I misunderstood something?

4. The authors indicate that a multivariable analysis was not done, but they do explain why. It leaves the analysis of factors associated with disease control open to a high probability of confounding bias, which cannot be addressed by a low degree of data “missingness and the data quality assurance procedures” in place (response of the authors)

Reviewer #2: no comment

**********

7. PLOS authors have the option to publish the peer review history of their article (what does this mean?). If published, this will include your full peer review and any attached files.

**Do you want your identity to be public for this peer review?**  If you choose “no”, your identity will remain anonymous but your review may still be made public.

For information about this choice, including consent withdrawal, please see our Privacy Policy.

Reviewer #1: No

Reviewer #2: No

**********

[NOTE: If reviewer comments were submitted as an attachment file, they will be attached to this email and accessible via the submission site. Please log into your account, locate the manuscript record, and check for the action link "View Attachments". If this link does not appear, there are no attachment files.]

-->Figure Resubmissions:

While revising your submission, we strongly recommend that you use PLOS’s NAAS tool (https://ngplosjournals.pagemajik.ai/artanalysis) to test your figure files. NAAS can convert your figure files to the TIFF file type and meet basic requirements (such as print size, resolution), or provide you with a report on issues that do not meet our requirements and that NAAS cannot fix.

After uploading your figures to PLOS’s NAAS tool - https://ngplosjournals.pagemajik.ai/artanalysis, NAAS will process the files provided and display the results in the "Uploaded Files" section of the page as the processing is complete. If the uploaded figures meet our requirements (or NAAS is able to fix the files to meet our requirements), the figure will be marked as "fixed" above. If NAAS is unable to fix the files, a red "failed" label will appear above. When NAAS has confirmed that the figure files meet our requirements, please download the file via the download option, and include these NAAS processed figure files when submitting your revised manuscript.

# Author comment: Author response to Decision Letter 2

**DOI:** 10.1371/journal.pgph.0006629.r004

3 Nov 2025

Attachment Submitted filename: Response to reviwers 2.docx

# Peer review: Decision Letter 2

**DOI:** 10.1371/journal.pgph.0006629.r005

**Authors:** Ari Probandari

28 Dec 2025

PGPH-D-25-00866R2

ASSESSING FACTORS ASSOCIATED WITH DISEASE CONTROL AMONG HYPERTENSIVE, DIABETIC, AND ASTHMATIC PATIENTS ENROLLED IN SECONDARY-LEVEL INTEGRATED CHRONIC CARE CLINICS IN NENO DISTRICT, RURAL MALAWI.

PLOS Global Public Health

Dear Dr. Limwado,

Thank you for submitting your manuscript to PLOS Global Public Health. After careful consideration, we feel that it has merit but does not fully meet PLOS Global Public Health’s publication criteria as it currently stands. Therefore, we invite you to submit a revised version of the manuscript that addresses the points raised during the review process.

Please submit your revised manuscript by Feb 11 2026 11:59PM. If you will need more time than this to complete your revisions, please reply to this message or contact the journal office at globalpubhealth@plos.org. When you're ready to submit your revision, log on to https://www.editorialmanager.com/pgph/ and select the 'Submissions Needing Revision' folder to locate your manuscript file.

Please include the following items when submitting your revised manuscript:

- A letter that responds to each point raised by the editor and reviewer(s). You should upload this letter as a separate file labeled 'Response to Reviewers'.
- A marked-up copy of your manuscript that highlights changes made to the original version. You should upload this as a separate file labeled 'Revised Manuscript with Track Changes'.
- An unmarked version of your revised paper without tracked changes. You should upload this as a separate file labeled 'Manuscript'.

Guidelines for resubmitting your figure files are available below the reviewer comments at the end of this letter.

We look forward to receiving your revised manuscript.

Kind regards,

Ari Probandari, PhD

Academic Editor

PLOS Global Public Health

Journal Requirements:

If the reviewer comments include a recommendation to cite specific previously published works, please review and evaluate these publications to determine whether they are relevant and should be cited. There is no requirement to cite these works unless the editor has indicated otherwise.

Additional Editor Comments (if provided):

[Note: HTML markup is below. Please do not edit.]

Reviewers' comments:

Reviewer's Responses to Questions

**Comments to the Author**

1. If the authors have adequately addressed your comments raised in a previous round of review and you feel that this manuscript is now acceptable for publication, you may indicate that here to bypass the “Comments to the Author” section, enter your conflict of interest statement in the “Confidential to Editor” section, and submit your "Accept" recommendation.

Reviewer #1: All comments have been addressed

Reviewer #3: (No Response)

**********

2. Does this manuscript meet PLOS Global Public Health’s publication criteria? Is the manuscript technically sound, and do the data support the conclusions? The manuscript must describe methodologically and ethically rigorous research with conclusions that are appropriately drawn based on the data presented.

Reviewer #1: No

Reviewer #3: No

**********

3. Has the statistical analysis been performed appropriately and rigorously?

Reviewer #1: No

Reviewer #3: No

**********

4. Have the authors made all data underlying the findings in their manuscript fully available (please refer to the Data Availability Statement at the start of the manuscript PDF file)?

The PLOS Data policy requires authors to make all data underlying the findings described in their manuscript fully available without restriction, with rare exception. The data should be provided as part of the manuscript or its supporting information, or deposited to a public repository. For example, in addition to summary statistics, the data points behind means, medians and variance measures should be available. If there are restrictions on publicly sharing data—e.g. participant privacy or use of data from a third party—those must be specified.

Reviewer #1: Yes

Reviewer #3: No

**********

5. Is the manuscript presented in an intelligible fashion and written in standard English?

PLOS Global Public Health does not copyedit accepted manuscripts, so the language in submitted articles must be clear, correct, and unambiguous. Any typographical or grammatical errors should be corrected at revision, so please note any specific errors here.

Reviewer #1: Yes

Reviewer #3: Yes

**********

6. Review Comments to the Author

Please use the space provided to explain your answers to the questions above. You may also include additional comments for the author, including concerns about dual publication, research ethics, or publication ethics. (Please upload your review as an attachment if it exceeds 20,000 characters)

Reviewer #1: See the attached document

Reviewer #3: Title and Abstract

The title implies a comprehensive assessment of factors associated with disease control, yet the quantitative analyses identify very few robust predictors, especially after multivariable adjustment. This creates a mismatch between the title and actual inferential strength of the results.

The abstract overstates conclusions regarding the “effectiveness” of the IC3 model, despite the absence of a comparator group or pre-post analysis to support effectiveness claims.

Introduction

While well-referenced, the introduction lacks a clear conceptual or analytical framework explaining how integrated care is hypothesized to influence disease control outcomes.

The research gap is framed broadly; however, the specific hypotheses tested in the quantitative analysis are not clearly articulated, making it difficult to assess whether the analyses are confirmatory or exploratory.

Methods

Study Design and Population

The retrospective cross-sectional design does not allow causal inference, yet the manuscript repeatedly implies causal relationships (e.g., medication number influencing control).

There are internal inconsistencies in dates (e.g., February-December 2022 vs July-December 2023 vs 2017-2021 enrollment), which raise concerns about data coherence and require clarification.

Disease Definitions and Measurement

Disease control is based on single most recent measurements, which may misclassify chronic control status and introduces measurement bias. This limitation is acknowledged but not sufficiently accounted for in interpretation.

Asthma control relies on self-reported symptoms without objective measures, increasing risk of reporting bias, particularly in qualitative-quantitative integration.

Sample Size and Power

Subgroup sizes for diabetes and COPD are very small, yet multivariable regression is performed. This raises serious concerns regarding model overfitting, unstable estimates, and wide confidence intervals.

Statistical Analysis

Variable selection for multivariable models is not justified (e.g., no explanation of inclusion criteria or handling of collinearity).

The number of predictors relative to outcome events, especially for diabetes and asthma models, violates recommended events-per-variable thresholds, undermining the reliability of adjusted odds ratios.

Several statistically significant univariate associations disappear in multivariable models, but this discordance is not critically discussed.

Results

Tables are dense and difficult to interpret, with limited narrative synthesis highlighting clinically meaningful findings.

The finding that “fewer medications” is associated with better control is likely confounded by disease severity, yet this alternative explanation is insufficiently explored.

COPD results are reported despite an extremely small sample (n=24), limiting interpretability and adding little value to the analysis.

Qualitative Component

While rich in quotations, the qualitative analysis lacks methodological depth:

No reflexivity statements

Limited description of coder training or intercoder reliability

Integration of qualitative and quantitative findings is largely descriptive, rather than analytically integrated to explain discordant or null quantitative results.

Discussion

The discussion overinterprets associations as indicators of IC3 effectiveness without appropriate comparative data.

Alternative explanations (e.g., survivorship bias, care-seeking behavior bias, severity confounding) are not adequately explored.

The policy implications are strong, but the evidence base presented does not fully support scaling recommendations.

Limitations

Although limitations are listed, they are not sufficiently weighted in the conclusions. Key methodological constraints, particularly cross-sectional design, measurement limitations, and small subgroup sizes, should more explicitly temper claims.

Data Availability and Ethics

The data availability statement imposes multiple access barriers. While ethically justified, the authors should clarify how this aligns with PLOS data-sharing expectations and whether metadata or analytic code can be shared.

**********

7. PLOS authors have the option to publish the peer review history of their article (what does this mean?). If published, this will include your full peer review and any attached files.

**Do you want your identity to be public for this peer review?**  If you choose “no”, your identity will remain anonymous but your review may still be made public.

For information about this choice, including consent withdrawal, please see our Privacy Policy.

Reviewer #1: No

Reviewer #3: No

**********

[NOTE: If reviewer comments were submitted as an attachment file, they will be attached to this email and accessible via the submission site. Please log into your account, locate the manuscript record, and check for the action link "View Attachments". If this link does not appear, there are no attachment files.]

-->Figure Resubmissions:

While revising your submission, we strongly recommend that you use PLOS’s NAAS tool (https://ngplosjournals.pagemajik.ai/artanalysis) to test your figure files. NAAS can convert your figure files to the TIFF file type and meet basic requirements (such as print size, resolution), or provide you with a report on issues that do not meet our requirements and that NAAS cannot fix.

After uploading your figures to PLOS’s NAAS tool - https://ngplosjournals.pagemajik.ai/artanalysis, NAAS will process the files provided and display the results in the "Uploaded Files" section of the page as the processing is complete. If the uploaded figures meet our requirements (or NAAS is able to fix the files to meet our requirements), the figure will be marked as "fixed" above. If NAAS is unable to fix the files, a red "failed" label will appear above. When NAAS has confirmed that the figure files meet our requirements, please download the file via the download option, and include these NAAS processed figure files when submitting your revised manuscript.

Attachment Submitted filename: PGPH-D-25-00866.R2 Review for authors.docx

Attachment Submitted filename: Reviewer Comments.docx

# Author comment: Author response to Decision Letter 3

**DOI:** 10.1371/journal.pgph.0006629.r006

11 Mar 2026

Attachment Submitted filename: Response_to_reviwers_2_auresp_3.docx

# Peer review: Decision Letter 3

**DOI:** 10.1371/journal.pgph.0006629.r007

**Authors:** Ari Probandari

26 Mar 2026

PGPH-D-25-00866R3

DISEASE CONTROL AND ITS CORRELATES AMONG HYPERTENSIVE, DIABETIC, AND ASTHMATIC PATIENTS ENROLLED IN SECONDARY-LEVEL INTEGRATED CHRONIC CARE CLINICS IN NENO DISTRICT, RURAL MALAWI.

PLOS Global Public Health

Dear Dr. Limwado,

Thank you for submitting your manuscript to PLOS Global Public Health. After careful consideration, we feel that it has merit but does not fully meet PLOS Global Public Health’s publication criteria as it currently stands. Therefore, we invite you to submit a revised version of the manuscript that addresses the points raised during the review process.

Please submit your revised manuscript by Apr 25 2026 11:59PM. If you will need more time than this to complete your revisions, please reply to this message or contact the journal office at globalpubhealth@plos.org. When you're ready to submit your revision, log on to https://www.editorialmanager.com/pgph/ and select the 'Submissions Needing Revision' folder to locate your manuscript file.

Please include the following items when submitting your revised manuscript:

- A letter that responds to each point raised by the editor and reviewer(s). You should upload this letter as a separate file labeled 'Response to Reviewers'.
- A marked-up copy of your manuscript that highlights changes made to the original version. You should upload this as a separate file labeled 'Revised Manuscript with Track Changes'.
- An unmarked version of your revised paper without tracked changes. You should upload this as a separate file labeled 'Manuscript'.

Guidelines for resubmitting your figure files are available below the reviewer comments at the end of this letter.

As the corresponding author, your ORCID iD is verified in the submission system and will appear in the published article. PLOS supports the use of ORCID, and we encourage all coauthors to register for an ORCID iD and use it as well. Please encourage your coauthors to verify their ORCID iD within the submission system before final acceptance, as unverified ORCID iDs will not appear in the published article. *Only*  the individual author can complete the verification step; PLOS staff *cannot*  verify ORCID iDs on behalf of authors.

We look forward to receiving your revised manuscript.

Kind regards,

Ari Probandari, PhD

Academic Editor

PLOS Global Public Health

Journal Requirements:

1. If the reviewer comments include a recommendation to cite specific previously published works, please review and evaluate these publications to determine whether they are relevant and should be cited. There is no requirement to cite these works unless the editor has indicated otherwise.

2. Please review your reference list to ensure that it is complete and correct. If you have cited papers that have been retracted, please include the rationale for doing so in the manuscript text, or remove these references and replace them with relevant current references. Any changes to the reference list should be mentioned in the rebuttal letter that accompanies your revised manuscript. If you need to cite a retracted article, indicate the article’s retracted status in the References list and also include a citation and full reference for the retraction notice.

Additional Editor Comments (if provided):

[Note: HTML markup is below. Please do not edit.]

Reviewers' comments:

Reviewer's Responses to Questions

**Comments to the Author**

1. If the authors have adequately addressed your comments raised in a previous round of review and you feel that this manuscript is now acceptable for publication, you may indicate that here to bypass the “Comments to the Author” section, enter your conflict of interest statement in the “Confidential to Editor” section, and submit your "Accept" recommendation.

Reviewer #1: All comments have been addressed

Reviewer #3: All comments have been addressed

**********

2. Does this manuscript meet PLOS Global Public Health’s publication criteria? Is the manuscript technically sound, and do the data support the conclusions? The manuscript must describe methodologically and ethically rigorous research with conclusions that are appropriately drawn based on the data presented.

Reviewer #1: Yes

Reviewer #3: Partly

**********

3. Has the statistical analysis been performed appropriately and rigorously?

Reviewer #1: I don't know

Reviewer #3: Yes

**********

4. Have the authors made all data underlying the findings in their manuscript fully available (please refer to the Data Availability Statement at the start of the manuscript PDF file)?

The PLOS Data policy requires authors to make all data underlying the findings described in their manuscript fully available without restriction, with rare exception. The data should be provided as part of the manuscript or its supporting information, or deposited to a public repository. For example, in addition to summary statistics, the data points behind means, medians and variance measures should be available. If there are restrictions on publicly sharing data—e.g. participant privacy or use of data from a third party—those must be specified.

Reviewer #1: Yes

Reviewer #3: No

**********

5. Is the manuscript presented in an intelligible fashion and written in standard English?

PLOS Global Public Health does not copyedit accepted manuscripts, so the language in submitted articles must be clear, correct, and unambiguous. Any typographical or grammatical errors should be corrected at revision, so please note any specific errors here.

Reviewer #1: Yes

Reviewer #3: Yes

**********

6. Review Comments to the Author

Please use the space provided to explain your answers to the questions above. You may also include additional comments for the author, including concerns about dual publication, research ethics, or publication ethics. (Please upload your review as an attachment if it exceeds 20,000 characters)

Reviewer #1: Most comments have been addressed and the manuscript is now nearly ready for publication in my view

I still have a number of minor points for improvement:

General: use the same number of decimals for percentages consistently throughout all sections of the manuscript

Abstract (Methods): describe which associations in: Associations…..logistic regression.

Methods: was the cuff size adapted to BMI, in particular, were appropriate cuffs used of obese and overweight individuals? This may have repercussions for the association of BMI with BP control. If the same cuff size was used for all, I suggest that this needs to be added to the limitations

Methods (Sample size): this sentence is a Result: “A total of…..with A

sthma.” I suggest placing it in the Results and add a description of the overlap of conditions among participants.

Methods (Participant recruitment): I suggest adding information about how the randomization was conducted.

Methods (Statistical analysis): please add some details on the methodology of the multivariable logistic regression analysis, for instance, was backward elimination used or otherwise?

Results (bivariate analysis): the point estimates with CI (not just the p-values) need to be provided with significant associations, also in the abstract

Results: each of the three following paragraphs are interpretations of results and should appear in the Discussion in my view:

“These mixed levels of health literacy help explain the quantitative findings, which showed no consistent association between demographic factors and disease control for asthma and diabetes. Instead, variation in knowledge and beliefs about disease mechanisms may influence adherence and symptom management beyond measured clinical variables.”

“Such barriers may contribute to delayed care-seeking or irregular follow-up. These accounts provide context for the quantitative finding that a substantial proportion of participants had uncontrolled disease, particularly among individuals with diabetes and hypertension. Travel burden and financial constraints may limit continuity of care, medication refills, and monitoring visits necessary for optimal disease control.”

“These narratives align with the quantitative findings showing associations between medication regimens and disease control, particularly for hypertension. Effective communication and counselling appear central to supporting adherence to both medication and lifestyle modifications.”

Discussion: please add to the limitations section that the probability of confounding is considerable in the analyses of factors associated with diabetes and asthma control, as the analyses did not correct for confounding statistically.

Reviewer #3: The manuscript has improved substantially and most major concerns have been addressed. The study is now methodologically sound, and interpretations are appropriately cautious. However, minor issues remain regarding clarity of study timelines, statistical model description, and data availability compliance.

**********

7. PLOS authors have the option to publish the peer review history of their article (what does this mean?). If published, this will include your full peer review and any attached files.

**Do you want your identity to be public for this peer review?**  If you choose “no”, your identity will remain anonymous but your review may still be made public.

For information about this choice, including consent withdrawal, please see our Privacy Policy.

Reviewer #1: No

Reviewer #3: No

**********

[NOTE: If reviewer comments were submitted as an attachment file, they will be attached to this email and accessible via the submission site. Please log into your account, locate the manuscript record, and check for the action link "View Attachments". If this link does not appear, there are no attachment files.]

Figure Resubmissions:

While revising your submission, we strongly recommend that you use PLOS’s NAAS tool (https://ngplosjournals.pagemajik.ai/artanalysis) to test your figure files. NAAS can convert your figure files to the TIFF file type and meet basic requirements (such as print size, resolution), or provide you with a report on issues that do not meet our requirements and that NAAS cannot fix.

After uploading your figures to PLOS’s NAAS tool - https://ngplosjournals.pagemajik.ai/artanalysis, NAAS will process the files provided and display the results in the "Uploaded Files" section of the page as the processing is complete. If the uploaded figures meet our requirements (or NAAS is able to fix the files to meet our requirements), the figure will be marked as "fixed" above. If NAAS is unable to fix the files, a red "failed" label will appear above. When NAAS has confirmed that the figure files meet our requirements, please download the file via the download option, and include these NAAS processed figure files when submitting your revised manuscript.

Attachment Submitted filename: PGPH-D-25-00866.R3 Review JvO.docx

# Author comment: Author response to Decision Letter 4

**DOI:** 10.1371/journal.pgph.0006629.r008

8 May 2026

Attachment Submitted filename: Response_to_reviwers_2_auresp_4.docx

# Peer review: Decision Letter 4

**DOI:** 10.1371/journal.pgph.0006629.r009

**Authors:** Ari Probandari

26 May 2026

DISEASE CONTROL AND ITS CORRELATES AMONG HYPERTENSIVE, DIABETIC, AND ASTHMATIC PATIENTS ENROLLED IN SECONDARY-LEVEL INTEGRATED CHRONIC CARE CLINICS IN NENO DISTRICT, RURAL MALAWI.

PGPH-D-25-00866R4

Dear Dr Limwado,

We are pleased to inform you that your manuscript 'DISEASE CONTROL AND ITS CORRELATES AMONG HYPERTENSIVE, DIABETIC, AND ASTHMATIC PATIENTS ENROLLED IN SECONDARY-LEVEL INTEGRATED CHRONIC CARE CLINICS IN NENO DISTRICT, RURAL MALAWI.' has been provisionally accepted for publication in PLOS Global Public Health.

Before your manuscript can be formally accepted you will need to complete some formatting changes, which you will receive in a follow up email. A member of our team will be in touch with a set of requests.

Please note that your manuscript will not be scheduled for publication until you have made the required changes, so a swift response is appreciated.

IMPORTANT: The editorial review process is now complete. PLOS will only permit corrections to spelling, formatting or significant scientific errors from this point onwards. Requests for major changes, or any which affect the scientific understanding of your work, will cause delays to the publication date of your manuscript.

If your institution or institutions have a press office, please notify them about your upcoming paper to help maximize its impact. If they'll be preparing press materials, please inform our press team as soon as possible -- no later than 48 hours after receiving the formal acceptance. Your manuscript will remain under strict press embargo until 2 pm Eastern Time on the date of publication. For more information, please contact globalpubhealth@plos.org.

Thank you again for supporting Open Access publishing; we are looking forward to publishing your work in PLOS Global Public Health.

Best regards,

Ari Probandari, PhD

Academic Editor

PLOS Global Public Health

***********************************************************

Reviewer Comments (if any, and for reference):

Reviewer's Responses to Questions

**Comments to the Author**

1. If the authors have adequately addressed your comments raised in a previous round of review and you feel that this manuscript is now acceptable for publication, you may indicate that here to bypass the “Comments to the Author” section, enter your conflict of interest statement in the “Confidential to Editor” section, and submit your "Accept" recommendation.

Reviewer #1: All comments have been addressed

Reviewer #3: All comments have been addressed

**********

2. Does this manuscript meet PLOS Global Public Health’s publication criteria? Is the manuscript technically sound, and do the data support the conclusions? The manuscript must describe methodologically and ethically rigorous research with conclusions that are appropriately drawn based on the data presented.

Reviewer #1: Yes

Reviewer #3: Yes

**********

3. Has the statistical analysis been performed appropriately and rigorously?

Reviewer #1: Yes

Reviewer #3: Yes

**********

4. Have the authors made all data underlying the findings in their manuscript fully available (please refer to the Data Availability Statement at the start of the manuscript PDF file)?

The PLOS Data policy requires authors to make all data underlying the findings described in their manuscript fully available without restriction, with rare exception. The data should be provided as part of the manuscript or its supporting information, or deposited to a public repository. For example, in addition to summary statistics, the data points behind means, medians and variance measures should be available. If there are restrictions on publicly sharing data—e.g. participant privacy or use of data from a third party—those must be specified.

Reviewer #1: Yes

Reviewer #3: Yes

**********

5. Is the manuscript presented in an intelligible fashion and written in standard English?

PLOS Global Public Health does not copyedit accepted manuscripts, so the language in submitted articles must be clear, correct, and unambiguous. Any typographical or grammatical errors should be corrected at revision, so please note any specific errors here.

Reviewer #1: Yes

Reviewer #3: Yes

**********

6. Review Comments to the Author

Please use the space provided to explain your answers to the questions above. You may also include additional comments for the author, including concerns about dual publication, research ethics, or publication ethics. (Please upload your review as an attachment if it exceeds 20,000 characters)

Reviewer #1: No further comments

Reviewer #3: The revised manuscript has improved substantially and addresses the major concerns raised previously.

**********

7. PLOS authors have the option to publish the peer review history of their article (what does this mean?). If published, this will include your full peer review and any attached files.

**Do you want your identity to be public for this peer review?**  If you choose “no”, your identity will remain anonymous but your review may still be made public.

For information about this choice, including consent withdrawal, please see our Privacy Policy.

Reviewer #1: No

Reviewer #3: No

**********
